# Dual mode imaging guided multi-functional bio-targeted oxygen production probes for tumor therapy

**DOI:** 10.1186/s12951-023-01901-7

**Published:** 2023-04-29

**Authors:** Yaotai Wang, Zhong Zhang, Li Ren, Yong Luo, Qi Wang, Jianzhong Zou

**Affiliations:** 1 State Key Laboratory of Ultrasound in Medicine and Engineering, College of Biomedical Engineering, Chongqing Medical University, Chongqing, 400016 China; 2 Chongqing Key Laboratory of Biomedical Engineering, Chongqing Medical University, Chongqing, 400016 China

**Keywords:** Bacteriotherapy, Tumor hypoxia, Focused ultrasound ablation surgery, Dual mode imaging, Anti-tumor therapy

## Abstract

Focused ultrasound ablation surgery (FUAS) is a novel therapy with a wide range of potential applications. However, synergists are crucial to the therapy process due to the ultrasonic energy's attenuation properties. As a result of the complex hypoxic environment in the tumor area and many factors, the existing synergists have limitations such as weak targeting, single imaging mode, and easy tumor recurrence after treatment. Because of the above deficiencies, this study intends to construct bio-targeted oxygen production probes consisting of *Bifidobacterium* that naturally target the hypoxia region of the tumor and multi-functional oxygen-producing nanoparticles equipped with IR780, perfluorohexane (PFH), CBP (carboplatin), and oxygen. The probes are expected to achieve targeted and synergistic FUAS therapy and dual-mode imaging to mediate tumor diagnosis and treatment. The oxygen and drugs carried in it are accurately released after FUAS stimulation, which is expected to alleviate tumor hypoxia, avoid tumor drug resistance, improve the effect of chemotherapy, and realize FUAS combined with chemotherapy antitumor therapy. This strategy is expected to make up for the deficiencies of existing synergists, improve the effectiveness and safety of treatment, and provide the foundation for future tumor therapy progress.

**Graphical Abstract:**

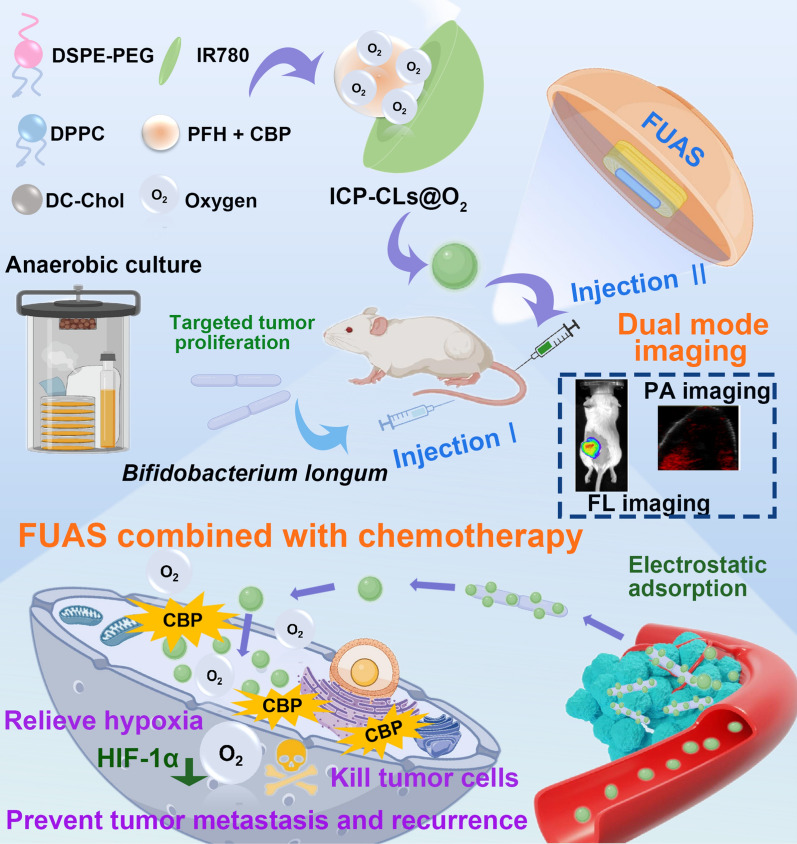

**Supplementary Information:**

The online version contains supplementary material available at 10.1186/s12951-023-01901-7.

## Background

Tumor precision therapy is a hot research topic at present [1, 2]. Focused ultrasound ablation surgery (FUAS), a new micro-non-invasive therapy technology, has been fully applied in treating tumors [3, 4]. FUAS can precisely kill tumor cells and achieve therapeutic effects through thermal, mechanical, and cavitation effects [5]. Due to the attenuation property of ultrasound, the efficacy of FUAS in the ablation of deep tumors and large tumors in vivo will be affected [6]. Traditional approaches to increasing the therapeutic effect and duration of ablation can improve treatment efficacy but can also result in worrying side effects such skin or nerve damage [7]. The researchers suggest that the situation could be improved by combining chemotherapy drugs with the use of synergists [8, 9]. However, hypoxic regions in the tumor and the limited targeting of existing synergists present a significant therapeutic challenge. As a typical feature of the solid tumor microenvironment, hypoxia, an unfavorable characteristic of the tumor tissue, is closely related to tumor resistance to several cancer therapies [10, 11]. Through continuous regulation of hypoxia-inducing factor-1α (HIF-1α) and multidrug resistance gene 1 (MDR 1), hypoxia often leads to chemotherapy failure and tumor metastasis [12, 13]. Therefore, the key to solving the problem is to select a suitable carrier to deliver oxygen and improve the oxygen-poor environment at the tumor site. It has been proposed that hypoxia can be relieved by hyperbaric oxygen inhalation [14], manganese dioxide or catalase as catalysts, or in situ oxygen production in the tumor microenvironment [15]. But these are not ideal solutions, concerns include the possibility of manganese toxicity, the lack of tumor selectivity in hyperbaric oxygen therapy, the efficiency of the treatment being constrained by hyperoxic toxicity, and the potential instability of catalase [16, 17]. PFH has attracted attention due to its characteristics. PFH has reliable biosafety and high solubility to oxygen and has been widely developed as a type of artificial blood substitute and oxygen carrier [18]. Although the oxygen release efficiency of PFH in conventional mode is limited only through oxygen concentration gradient diffusion, focused ultrasound with good tissue penetration can accurately control and stimulate the oxygen release of PFH [19]. The thermal effect, on the other hand, can raise the temperature of the tumor, increasing blood perfusion and velocity and thus relieving hypoxia [20, 21]. At the same time, perfluorohexane (PFH) is encapsulated in nanoparticles (NPs) and stimulated by ultrasound as a unique liquid–gas phase-shifting substance [22], which can transform phase into microbubbles and act as cavitation nuclei to enhance the effectiveness of FUAS [23, 24]. Therefore, selecting NPs containing PFH as oxygen carriers and synergistic substances will kill two birds with one stone, enhance FUAS and relieve hypoxia [25, 26].

Another critical problem is the targeting of PFH NPs. The lack of targeting of NPs will disperse throughout the body, and the amount of retention in tumor target areas will be small, which will affect the efficacy and safety of treatment. Although tumor targeting can be achieved by labeling specific proteins, the wide variety of tumors and the microenvironment in vivo reduce the targeting ability of NPs [27]. Given the nature of solid tumors, the presence of hypoxic areas and bacterial vectors provide a new direction for targeted therapy [28–30]. Obligate or facultative anaerobic bacteria represented by *Bifidobacterium* and *Escherichia coli*, due to their unique physiological characteristics, not only have specific targeting to solid tumors but also can be used as vectors for tumor-targeted therapy [31–33] and can realize antitumor therapy [34–37]. It is noted that *bifidobacterium*, as a probiotic, is the best carrier for tumor therapy due to its biological safety. The *bifidobacterium* has a negative surface charge. Without altering its physiological properties, *bifidobacterium* is anticipated to use the electrostatic adsorption to direct cationic NPs to assemble in the tumor target area and achieve biological targeting [38–40].

With the increase of clinical demand, a single synergist cannot meet the increasing clinical demand, so the construction of NPs is becoming increasingly multi-functional. Diagnosis and treatment of tumors are closely related to imaging. While a single imaging mode cannot provide comprehensive image information, multimodal imaging will compensate for the deficiency of single imaging and provide comprehensive diagnosis and treatment information, which is of great significance to clinical treatment [41–43]. IR780, a lipophilic cationic compound, has outstanding fluorescence (FL) and photoacoustic (PA) imaging ability. Without modification of ligands, IR780 itself has specific tumor-targeting properties, making it an ideal imaging dye [44, 45]. Compared with ultrasound imaging commonly used in FUAS image monitoring, FL and PA imaging have high sensitivity and high resolution, which can observe tumor target areas and evaluate the dynamic distribution of NPs in vivo, effectively making up for the shortcomings of existing imaging.

In addition to imaging monitoring, chemotherapy drugs used in combination therapy are also important. Carboplatin (CBP), a platinum analog, is a second-generation platinum-based drug. Currently, it is widely used by FDA to treat various types of cancer with chemotherapy and has an excellent anti-tumor effect due to its low cytotoxicity [46–48]. The introduction of chemotherapy drugs will reduce the possibility of tumor recurrence when FUAS ablation is incomplete [32].

**Scheme 1.**
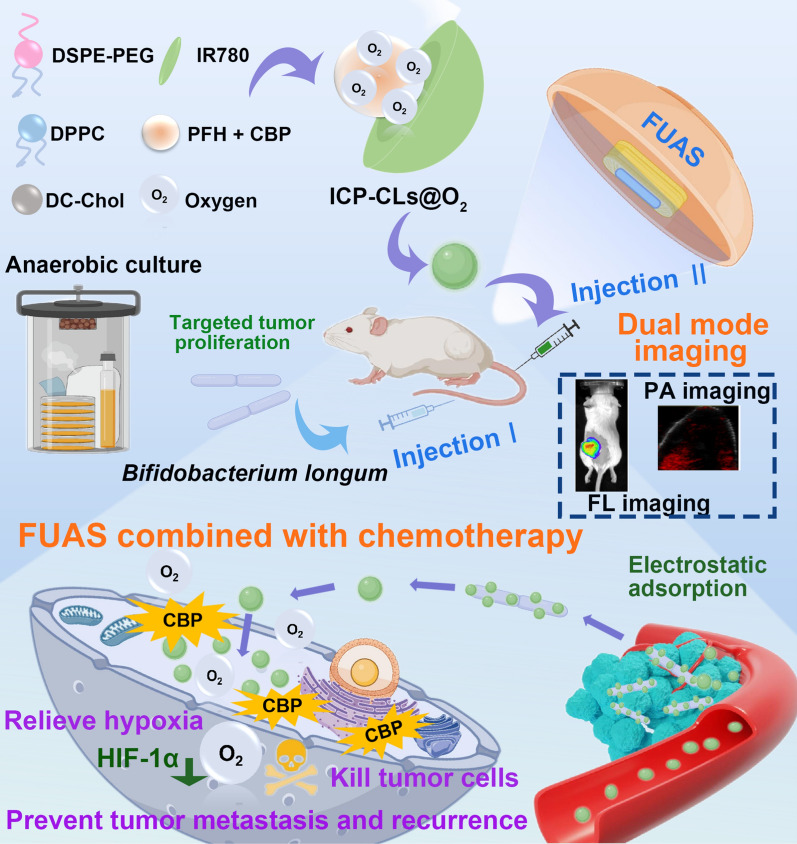
Dual mode imaging guided multi-functional bio-targeted oxygen production probes for tumor therapy

To summarize, this study developed bio-targeted oxygen production probes in response to the limitations of existing tumor therapy, such as poor targeting, a single diagnosis and treatment image mode, and drug resistance caused by hypoxia. As illustrated in Scheme 1, the probe mainly comprises biologic targeting vector *bifidobacterium* and multi-functional oxygen-producing NPs equipped with IR780, PFH, CBP, and O_2_ (ICP-CLs@O_2_). In order to avoid the influence of chemical modification on the physiological characteristics of *bifidobacterium* and the impact of excessive particle size on the delivery efficiency, we chose a two-step delivery method in terms of delivery mode. Firstly, *bifidobacterium* was injected to proliferate at the tumor target area, and then multi-functional oxygen-producing NPs (ICP-CLs@O_2_) were injected. With the guidance of the electrostatic adsorption force, the self-assembly of the bio-targeted oxygen production probes in the tumor target area was completed. Under the constant monitoring of bio-targeted oxygen production probes dual-mode imaging, targeted synergistic FUAS therapy can be realized, and chemotherapy drugs and oxygen can be released at a specific point to improve tumor resistance caused by hypoxia. Finally, FUAS combined with chemotherapy can be realized as antitumor therapy and inhibit tumor growth and metastasis. This strategy is expected to compensate for tumor therapy's shortcomings and has a potential application prospect.

## Materials and methods

### Materials and reagents

*Bifidobacterium longum* (ATCC 15707) was obtained from the Chongqing Key Laboratory of Biomedical Engineering. Man-Rogosa-Sharpe (MRS) broth was purchased from Qingdao Haibo Biotechnology Co. (Qingdao, China). 4,6-Diamidino-2-phenylindole (DAPI), DiI, Fluorescein isothiocyanate (FITC), and Cell Counting Kit-8 (CCK-8) assay were obtained from Beyotime Technology. (China). Chongqing East Chemical Industry Ltd., Co. (Chongqing, China) provided the chloroform (CHCl_3_). It was obtained from Avanti Polar Lipids in Alabama (USA) that DC-cholesterol hydrochloride (DC-CHOL), DSPE-PEG 2000-Amine, and DPPC were manufactured. IR780, perfluorohexane (PFH), and Carboplatin (CBP) were purchased from Aladdin Co., Ltd.

### *Bifidobacterium longum* (ATCC 15707) culture

*Bifidobacterium longum* (*BL*) was cultured anaerobically at 37 °C in MRS broth until the mid-log phase of growth. *BL* was collected and resuspended in PBS in the cryogenic centrifuge and adjusted to the appropriate reserve concentration (4000 rpm, 10 min, 4 °C).

### Synthesis of multi-functional oxygen-producing nanoparticles

A simple one-step emulsion procedure synthesized multi-functional oxygen-producing nanoparticles (NPs) [44]. The liposome film consists of DPPC, DC-cholesterol, DSPE-PEG2000, and IR780 mixed in a mass ratio of 12:4:4:1. The mixed drug and lipids were weighed according to the corresponding mass ratio and dissolved in CHCl_3,_ rotated on a rotary evaporator (Yarong Inc., Shanghai, China) at 50 °C to obtain a liposome film containing IR780 after removing the organic solvent. To completely dissolve the lipid film, deionized (DI) water was added. Under ice-water bath conditions, the mixed solution was transferred to an EP tube, and 200 µL of PFH and CBP were added and sonicated at 105W for 5 min (Sonics & Materials, Inc., USA). The unwrapped drug was removed by centrifugation at 8000 rpm (5 min, 4 ℃). The NPs were suspended in DI water, and the steps were repeated to purify to obtain multi-functional cationic lipid nanoparticles loaded with IR780, CBP, and PFH (ICP-CLs). At last, multi-functional oxygen-producing NPs (ICP-CLs@O_2_) could be harvested by bubbling oxygen into ICP-CLs for 5 min. DC-cholesterol was replaced with cholesterol to synthesize ICP-Ls@O_2_. Fluorescent NPs can be obtained by adding dye DiI into the lipid solution.

### Characterization of *BL* and multi-functional oxygen-producing nanoparticles

The morphology of *Bifidobacterium longum* was observed under a light microscope after gram staining. Using the NanoBrook 90Plus PALS (Brookhaven), the mean particle sizes and zeta potential of *Bifidobacterium longum* and ICP-CLs@O_2_ were tested. Transmission electron microscopy (TEM) was used to observe the internal structure of ICP-CLs@O_2_. With the UV–vis-NIR spectrophotometer (Shimadzu, Japan), the absorbance of ICP-CLs@O_2,_ CBP, and IR780 was measured, and standard curves were plotted for CBP and IR780. The oxygen concentration was measured using a portable dissolved oxygen meter (JPB-607A, Shanghai) as the primary testing medium for the oxygen loading and release behavior in aqueous solutions. In order to pre-oxygenate ICP-CLs@O_2_, ICP-CLs were saturated with pure oxygen. To verify the effect of FUAS on drug release, the in vitro release of CBP from ICP-CLs@O_2_ was detected under different conditions. As a test of biocompatibility for ICP-CLs@O_2_, hemolysis was performed. Multi-functional oxygen-producing NPs stability was tested every four days by measuring particle size and zeta potential, which laid the foundation for follow-up in vivo testing.

### Construction of the Bio-targeted oxygen production probes

In order to construct the Bio-targeted oxygen production probes, *BL* (1 × 10^5^ CFU/mL) and ICP-CLs@O_2_ (0.2 mg/mL) were mixed at a volume ratio of 1:3, then fully reacted at room temperature and let stand. The zeta potential and particle size of bio-targeted oxygen production probes were further detected.

To further test the connection between *BL* and ICP-CLs@O_2_, verify whether bio-targeted oxygen production probes are successfully constructed. The ICP-Ls@O_2_ and ICP-CLs@O_2_ were labeled with DiI, and *BL* was labeled with FITC. Bio-targeted group (*BL* + ICP-CLs@O_2_) and non-targeted group (*BL* + ICP-Ls@O_2_) were observed via confocal laser scanning microscopy (CLSM, Nikon A1, Japan), judging the conglutination between *BL* and NPs. Flow cytometry (FCM, BD FACSVantage SE, USA) was used to compare the bind efficiencies of the bio-targeted and non-targeted groups.

### Cell culture and animal mode

Murine breast cancer 4T1 cells and human umbilical vein endothelial cells (HUVECs) were provided by the Chinese Academy of Science Cell Bank, by the American Type Culture Collection (ATCC) recommendations at 37 ℃ under 5% CO_2_.

All animal treatments approved by the Chongqing Medical University Animal Ethics Committee were guided by the Chongqing Medical University Guidelines for the Care and Use of Laboratory Animals. Female BALB/c mice (6–8 weeks) were purchased from Chongqing Medical University's Laboratory Animal Center. 4T1 cells were injected subcutaneously into the mice's left flank with 1 × 10^6^ cells suspended in 100 µL PBS solution to create the 4T1 tumor model. When the tumor diameter reached 0.5 cm, in vivo experiments were carried out.

### In vitro chemotherapy of ICP-CLs@O_2_

4T1 cells was inoculated into a confocal dish for 12 h and divided into the ICP-CLs@O_2_ group and ICP-CLs@O_2_ + FUAS group. After cell proliferation was stabilized, ICP-CLs@O_2_ irradiated by FUAS were added to the ICP-CLs@O_2_ + FUAS group, and an equal dose of ICP-CLs@O_2_ without FUAS was added to ICP-CLs@O_2_ group. In order to distinguish between live and dead cells, both groups of cells were stained with calcein-AM and observed using CLSM. Flow cytometry further quantified the cell apoptosis rate of the two groups and evaluated the chemotherapy effect of ICP-CLs@O_2_ on tumor cells.

### Targeted detection of bio-targeted oxygen production probes

Fifteen tumor-bearing mice were randomly divided into the Control group, post-3-day group, and post 7-day group. The mice in the last two groups were given *BL* (200 µL, 1 × 10^6^ CFU/mL) injections through the tail vein for three consecutive days. In the control group, mice were injected with 200 µL PBS. At post-three and post-seven days after injection, the mice in the corresponding group were euthanized, and the tumor, heart, liver, spleen, kidney, and lung tissues were extracted and homogenized. The tissue homogenates, after serially diluted, were cultured anaerobically on solid agar for 48 h. The distribution of *BL *in vivo tissues was assessed by observing the growth condition of colonies on the agar.

30 tumor-bearing mice were randomly divided into a bio-targeted group and a non-targeted group. In the bio-targeted group, mice were injected *BL* (1 × 10^6^ CFU/mL, 200 µL) intravenously for three consecutive days. The mice in the non-targeted group were injected with 200 µL PBS similarly. At 7 days after injection, both groups were inject 200 µL DiI-ICP-CLs@O_2_ (1 mg/mL) intravenously. Mice were euthanized in two groups 24 h, 48 h, and 72 h after the DiI-ICP-CLs@O_2_ injection. Tumor tissue was extracted and prepared into frozen ultrathin slices. The tumor nuclei of frozen ultrathin slices were labeled with DAPI and scanned via CLSM to evaluate the targeting ability of bio-targeted oxygen production probes.

### Evaluation of the ability to relieve hypoxia in vivo

HypoxyprobeTM-1 Kit was used to evaluate the hypoxia situations in the PBS group, ICP-CLs group, ICP-CLs@O_2_ group, and *BL* + ICP-CLs @O_2_ group (bio-targeted oxygen production probes group). Tumor-bearing mice in the bio-targeted oxygen production probes group were i.v. Injected with 200 µL *BL* (1 × 10^6^ CFU/mL) three times. Mice in the PBS group, ICP-CLs group, and ICP-CLs@O_2_ group were i.v. Injected with the same dose of PBS. Seven days after injection, the corresponding group was injected with PBS, ICP-CLs, and ICP-CLs@O_2,_ respectively. 48 h after the second injection, FUAS treatment was administered. Then mice were given pimonidazole hydrochloride (60 mg/kg) intraperitoneally. Tumors were harvested and prepared into frozen ultrathin slices thirty minutes later. In the process of immunofluorescence staining, the mouse monoclonal antibody was used for incubation first, followed by a Dylight 649-conjugated goat anti-mice secondary antibody. In order to locate the nucleus clearly, the nuclei of tumor cells were stained with DAPI.

HIF-1α expression was related to the degree of hypoxia. The experimental grouping and treatment were the same as above. The mice were sacrificed after the treatment, and the tumor tissues were extracted for the frozen section. HIF-1α rabbit antibody was used as the primary immunostain, followed by FITC-conjugated AffiniPure goat anti-rabbit secondary antibody, following DAPI staining of the tumor cell nuclei.

All fluorescence images were observed using CLSM.

### Dual-modality imaging in vitro

To test the fluorescence (FL) imaging capability of multi-functional oxygen-producing nanoparticles, 200 μL of samples with CP-CLs@O_2_ and different concentrations of ICP-CLs@O_2_ were tested. The LB983 imaging system was used to capture the FL imaging (Berthold Technologies GmbH & Co. KG, Germany).

The agar gel model was supplemented with various concentrations of ICP-CLs@O_2_ to identify the photoacoustic (PA) imaging capability. The Vevo LAZR PA Imaging System was used to obtain the PA imaging (Visual Sonics Inc., Toronto, Canada).

### Dual-modality imaging in vivo

For in vivo dual-modality imaging, mice were randomly divided into bio-targeted group and non-targeted groups with five mice each. First, mice in the bio-targeted group were injected with *BL* (200 µL, 1 × 10^6^ CFU/mL) intravenously for three consecutive days. Mice in the non-targeted group were treated with the same dose of PBS. Seven days after injection, mice in both groups were injected with 200 µL ICP-CLs@O_2_ through the tail vein. PA and FL Images of mice in two groups were captured at various time points after injection. To further observe the biological distribution of the bio-targeted oxygen production probes in mice, tumors and major organs of mice were extracted for FL imaging.

### Synergistic effect of bio-targeted oxygen production probes with FUAS

Fresh ex vivo bovine livers were used to test the in vitro synergistic capability of multi-functional oxygen-producing nanoparticles.100 μL PBS, ICP-CLs, and ICP-CLs@O_2_ were injected into bovine livers separately. Guided by ultrasonic images, technicians located the target area. The bovine livers target area was then treated for 3 s at 150 W with a Model-JC200 Focused Ultrasound Tumor Therapeutic System (Chongqing Haifu Medical Technology Co., Ltd., Chongqing, China). The ultrasonic images of the target area before and after treatment were sketched, and the gray change values were calculated using Gray Val software associated with the FUAS equipment. Bovine liver after ablation with focused ultrasound was sliced to determine the maximal cross-section of the ablation area and calculate the volume of coagulated necrosis.

PBS group, ICP-CLs group, ICP-CLs@O_2_ group, and *BL* + ICP-CLs@O_2_ group with five mice each to evaluate in vivo synergistic capability of FUAS bio-targeted oxygen production probes. For the first injection, mice in the *BL* + ICP-CLs@O_2_ group were i.v. injected with *BL* (200 µL, 1 × 10^6^ CFU/mL) for three consecutive days. Mice in other groups were i.v. injected with PBS. For the second injection, 200 μL PBS, ICP-CLs, and ICP-CLs@O_2_ were i.v. injected into the corresponding groups. FUAS was administered under ultrasound guidance 48 h after injection. The ultrasonic images of the tumor target area before and after treatment were sketched, and the gray change values were calculated. Twenty-four hours after FUAS, mice were euthanized, and tumors were harvested to evaluate the coagulative necrosis degree of the tumor more intuitively. The tumor tissue was incised along the acoustic beam axis to find the maximum ablation section, and TTC staining was performed (37 ℃, 30 min). The formula calculated coagulation necrosis volume and energy efficiency factor (EEF):

$$\mathrm{V}(\mathrm{mm}^3= (\uppi /6) \times \mathrm{ length }\times \mathrm{ width }\times \mathrm{ depth};\mathrm{ EEF}(\mathrm{J}/{\mathrm{mm}}^{3}) =\mathrm{ \eta Pt}/\mathrm{V}(\upeta = 0.7,\mathrm{ P}=150\,\mathrm{ W},\mathrm{ t}=3\,\mathrm{s})$$

### FUAS-chemo combined antitumor therapy in vivo

The mice experimental grouping and treatment process was the same as in vivo synergistic experience. After the first injection, tumor volume was measured every four days. To evaluate the relative tumor volume (RTV) before and after treatment, the initial tumor volume before the first injection was used as standardized. In order to analyze necrosis and apoptosis, mice were euthanized 17 days after injection. The tumor samples were collected and weighed before staining with H&E, PCNA, and TUNEL to evaluate the effect of antitumor therapy in vivo.

### Biosafety assessment

The cytotoxicity of ICP-CLs@O_2_ against normal HUVECs was evaluated using the Cell Counting Kit-8 (CCK-8) assay. Blood samples were collected short, and long-term after being treated with bio-targeted oxygen production probes were injected and then detected for biochemical tests (alanine aminotransferase, ALT; aspartate transaminase, AST; CK; blood urea nitrogen, BUN) and complete blood count (white blood cell, WBC; red blood cell, RBC; hemoglobin, HGB; platelets, PLT) to check the biosecurity. H&E staining was performed on major organs of mice after combination therapy to evaluate the safety of the treatment.

### Statistical analysis

Data were expressed as mean and standard deviation (SD) and statistically analyzed using GraphPad Prism 9.2.0. Student's t-test was used to identify the statistical significance between the two groups. One-way analysis of variance (ANOVA) was applied for multiple group comparisons. *P* (*) < 0.05 indicated statistical significance.

## Results and discussions

### Characterization of ICP-CLs@O_2_ and ***Bifidobacterium longum***

**Fig. 1:**
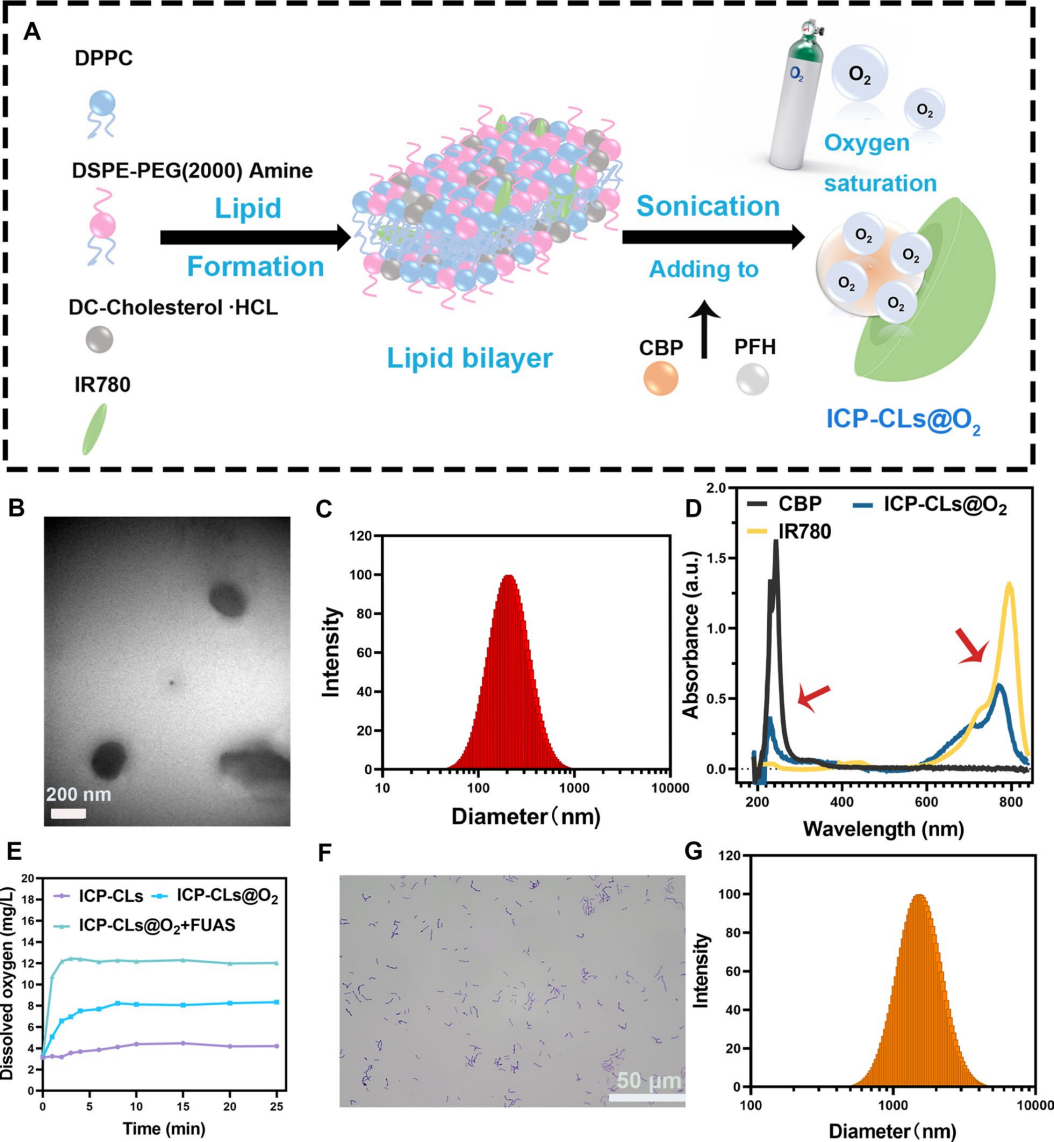
Characterization of ICP-CLs@O_2_ and *Bifidobacterium longum*. **A** Synthetic process for ICP-CLs@O_2_. **B** TEM image of ICP-CLs@O_2_. **C** Size distribution of ICP-CLs@O_2_. **D** Absorbance spectra of CBP, IR780, and ICP-CLs@O_2_. **E** The O_2_ release curves of ICP-CLs@O_2_ with or without FUAS. **F** Gram staining of *Bifidobacterium longum*. **G** Size distribution of *Bifidobacterium longum*

According to the schematic diagram (Fig. 1A), a simple film hydration emulsification procedure is used to create ICP-CLs@O_2_. ICP-CLs@O_2_ were clearly spherical, with black and dense cores, according to the TEM results (Fig. 1B). Under a light microscope (Additional file 1: Fig. S1), the ICP-CLs@O_2_ showed good dispersion, and the particle size was 207.7 ± 4.04 nm (Fig. 1C), which was consistent with the results of TEM. CBP and IR780 each had specific absorption peaks at 229 nm and 780 nm. The UV spectra (Fig. 1D) showed that the ICP-CLs@O_2_ had specific absorption peaks at both places, indicating that the NPs were successfully loaded with CBP and IR780. The entrapment and loading efficiency of IR780 and CBP were calculated as 82.7%, 3.76wt% and 47.8%, 2.17wt%, respectively, using the standard curves of IR780 and CBP (Additional file 1: Figs. S2, S3). With the addition of ICP-CLs@O_2_ and ICP-CLs@O_2_ + FUAS, the concentration of dissolved oxygen increases, indicating that ICP-CLs@O_2_ has a good oxygen-carrying capacity (Fig. 1E). Due to the full van der Waals interactions between PFH and oxygen molecules, PFH may effectively dissolve oxygen gas [23, 49]. The oxygen concentration in group ICP-CLs@O_2_ + FUAS increased more rapidly than in group ICP-CLs@O_2_, where without FUAS stimulation, oxygen was released slowly. The results showed that FUAS stimulated the release of oxygen. This sudden release may be due to an increase in temperature due to the FUAS-induced thermal effect, which accelerates the evaporation of PFH and the corresponding oxygen release [50]. CBP in vitro release experiments showed that FUAS irradiation could better promote drug release (Additional file 1: Fig. S4), and the effect of local chemotherapy could be significantly enhanced under the action of FUAS irradiation, while avoiding adverse systemic side effects. After gram staining, *BL* showed a long purple rod shape under the light microscope, consistent with the typical characteristics of gram-positive bacteria (Fig. 1F). The particle size of *BL* was 1511.33 ± 12.22 nm (Fig. 1G). ICP-CLs@O_2_ have slight variations in particle size and zeta potential (Additional file 1: Figs. S5, S6), indicating long-term stability. The hemolysis experiment results showed that the hemolysis rates were all within the safe range after the addition of ICP-CLs@O_2_ with different concentrations (Additional file 1: Fig. S7). The ICP-CLs@O_2_ had good biocompatibility, which laid the foundation for subsequent in vivo experiments.

### Construction of the bio-targeted oxygen production probes

**Fig. 2:**
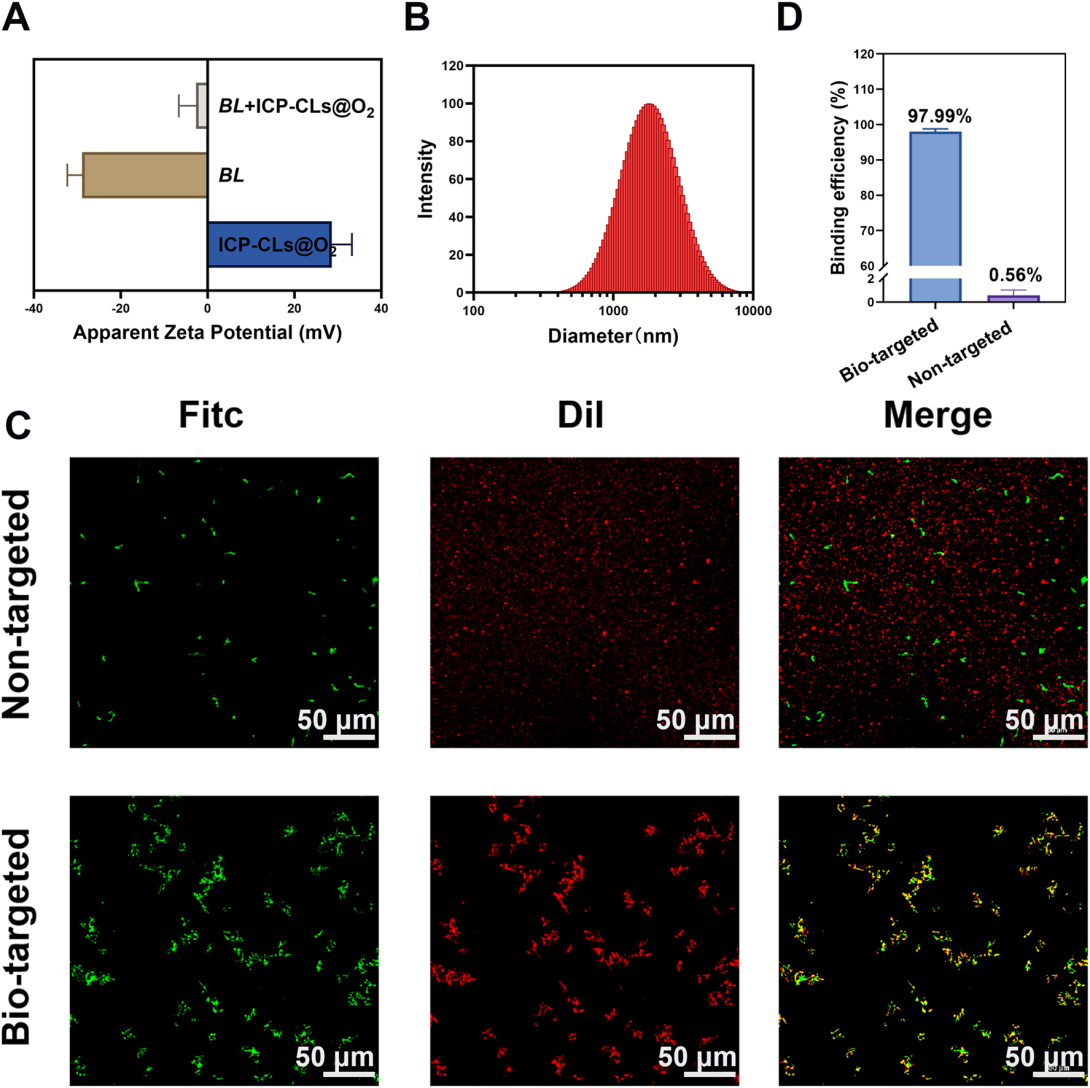
Construction of the Bio-targeted oxygen production probes. **A** Surface zeta potential of ICP-CLs@O_2_, *BL*, and Bio-targeted oxygen production probes. **B** Size distribution of *BL* + ICP-CLs@O_2_. **C** CLSM images of *BL* + ICP-CLs@O_2_ (Bio-targeted oxygen production probes) and *BL* + ICP-Ls@O_2_. **D** Statistical analysis of binding efficiency between *BL* and ICP-CLs@O_2_

The potential detection results showed that the apparent zeta potential of *BL* and ICP-CLs@O_2_ was about − 28.93 ± 3.38 mV and + 28.55 ± 4.68 mV, respectively (Fig. 2A). The potentials of ICP-CLs@O_2_ and *BL* were positive and negative, which have the potential for electrostatic adsorption. The bio-targeted oxygen-producing probe was an ICP-CLs@O_2_ and *BL* mixture with an apparent zeta potential of − 2.67 ± 3.98 mV and particle size of 1812.19 ± 19.38 nm (Fig. 2B). In order to more intuitively detect whether ICP-CLs@O_2_ and *BL* are successfully connected through electrostatic adsorption. *BL* was stained green by FITC, and the NPs were labeled red by DiI under CLSM. In the bio-targeted group, a large number of ICP-CLs@O_2_ adhered around *BL*, and their fluorescence fusion was orange. In the non-targeted, there was no combination of *BL* and ICP-Ls@O_2_ and no fluorescence fusion (Fig. 2C). Because the surface potential of ICP-CLs@O_2_ and ICP-Ls@O_2_ was different. ICP-Ls@O_2_ does not contain DC-Chol, so the surface potential was negative, which is consistent with *BL*, so it cannot be connected by electrostatic adsorption. This phenomenon also demonstrated that the bio-targeted oxygen production probes were successfully built using electrostatic adsorption. Flow cytometry further quantified the connection rate of the two (Additional file 1: Fig. S8). The connection rate of the bio-targeted group was 97.99%, 175 times higher than the non-targeted group (Fig. 2D). As a physical connection method, electrostatic adsorption avoids the change of *BL*. It retains its physiological characteristics by modifying the surface charge of NPs, which paves the way for subsequent in vivo targeting treatments.

### In vitro chemotherapy of ICP-CLs@O_2_

**Fig. 3:**
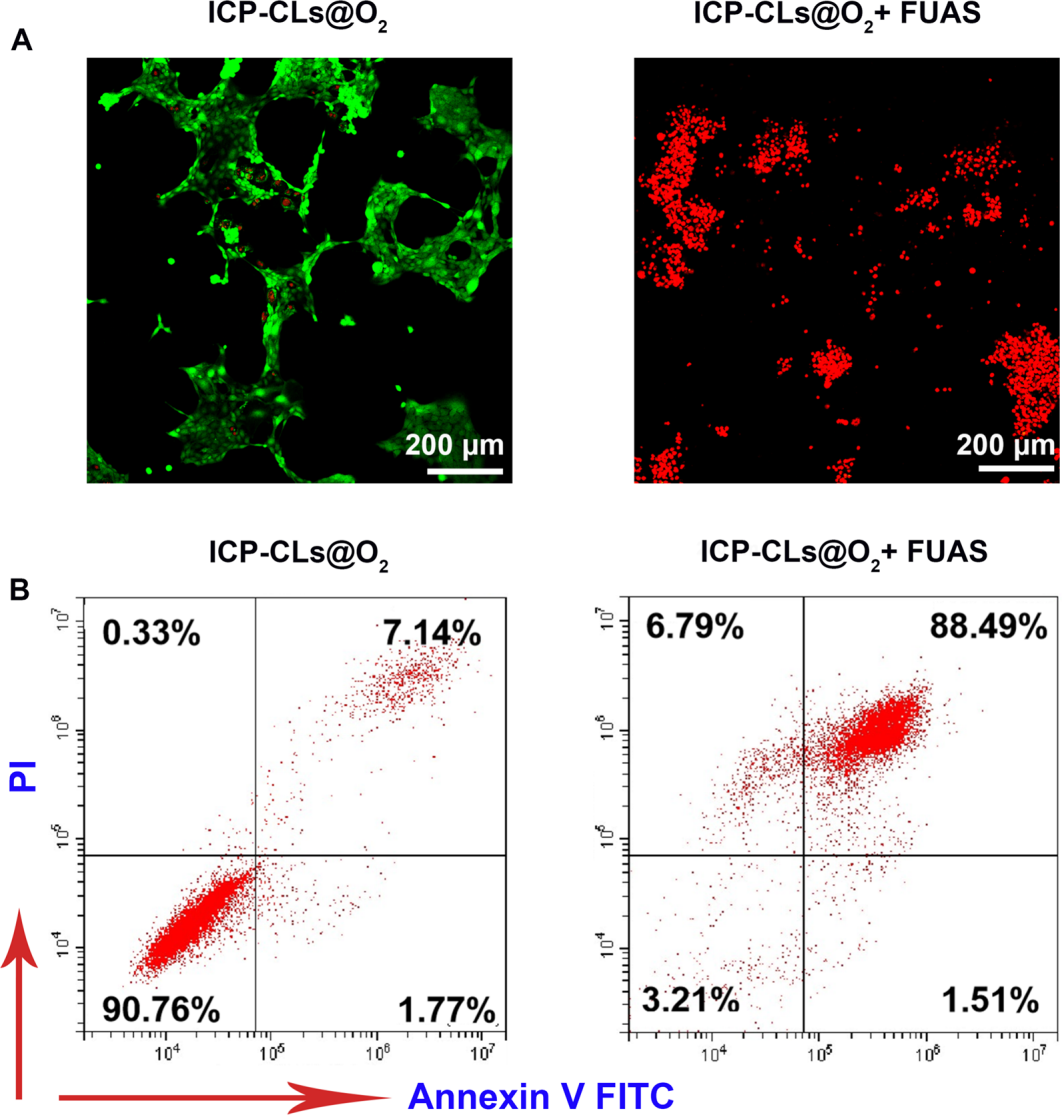
In vitro chemotherapy of ICP-CLs@O_2_. **A** Different treatment groups of CLSM image with calcein-AM and PI, living cells were labeled green, and dead cells were labeled red. **B** FCM apoptosis results for ICP-CLs@O_2_ and ICP-CLs@O_2_ + FUAS

After CAM-PI staining, the cells under different treatment conditions showed different results. Under CLSM, living cells were labeled green, and dead cells were labeled red. A large number of dead cells could be seen in the visual field of the ICP-CLs@O_2_ + FUAS group compared to the ICP-CLs@O_2_ group (Fig. 3A). The apoptosis rate of the ICP-CLs@O_2_ + FUAS group was as high as 88.49%, while that of the ICP-CLs@O_2_ group was only 7.14% (Fig. 3B), indicating that the drug released in the ICP-CLs@O_2_ could effectively kill tumor cells after stimulated by FUAS. When CBP is contained in NPs, it is only released at a predetermined location when activated by FUAS, minimizing harm to healthy tissue and enhancing the efficacy of chemotherapy.

### Targeted detection of bio-targeted oxygen production probes

**Fig. 4:**
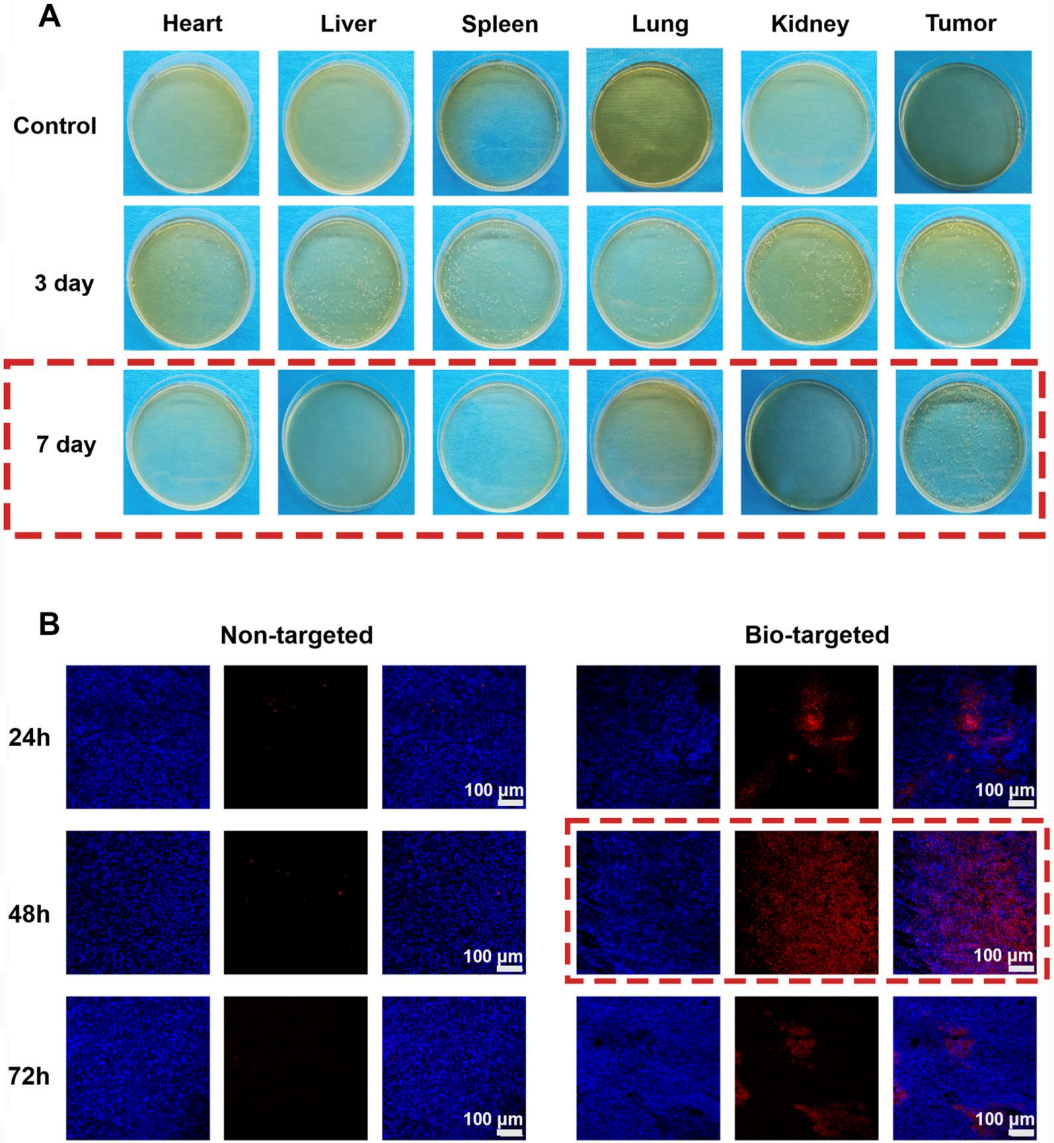
Targeted detection of bio-targeted oxygen production probes **A** The colonization of *BL* in various organs on agar plates **B** CLSM image of ultrathin section of tumor tissues at 24 h, 48 h, 72 h after injection of ICP-CLs@O_2_ and *BL* + ICP-CLs@O_2_ (Bio-targeted oxygen production probes)

After homogenization, the number of colonies determined the distribution of *BL* in the tissue on the agar plate. As shown in Fig. 4A, on the control group's plate, there were no bacterial colonies to be found. Following *BL* injection, the bacterial colonies of the *BL* group steadily developed over time at tumor sites while gradually declining in other tissues and organs. This phenomenon suggests that *BL* is first distributed throughout the body with the blood and then progressively removed by the body. Because the hypoxic environment inside the tumor promotes the growth of anaerobic *BL*, it colonizes the tumor target region. This trend is consistent with previous literature, *BL* can be used as a tumor-targeting vector [30].

ICP-CLs@O_2_ were marked with DiI to confirm the bio-targeted oxygen production probe’s targeting of the tumor (Fig. 4B). In contrast to the non-targeted group without *BL* injection, a significant number of ICP-CLs@O_2_ persisted in the tumor target region of the bio-targeted group. The electrostatic adsorption force can direct ICP-CLs@O_2_ to aggregate in the tumor target area because *BL* colonizing in the tumor target area can be employed as a target. By trapping the ICP-CLs@O_2_, bio-targeted oxygen production probes can then self-assemble in the tumor target area to achieve tumor targeting. The tumor-specific targeting of the bio-targeted oxygen production probes is crucial for subsequent in vivo synergism and antitumor therapy.

### Evaluation of the ability to relieve hypoxia in vivo

**Fig. 5:**
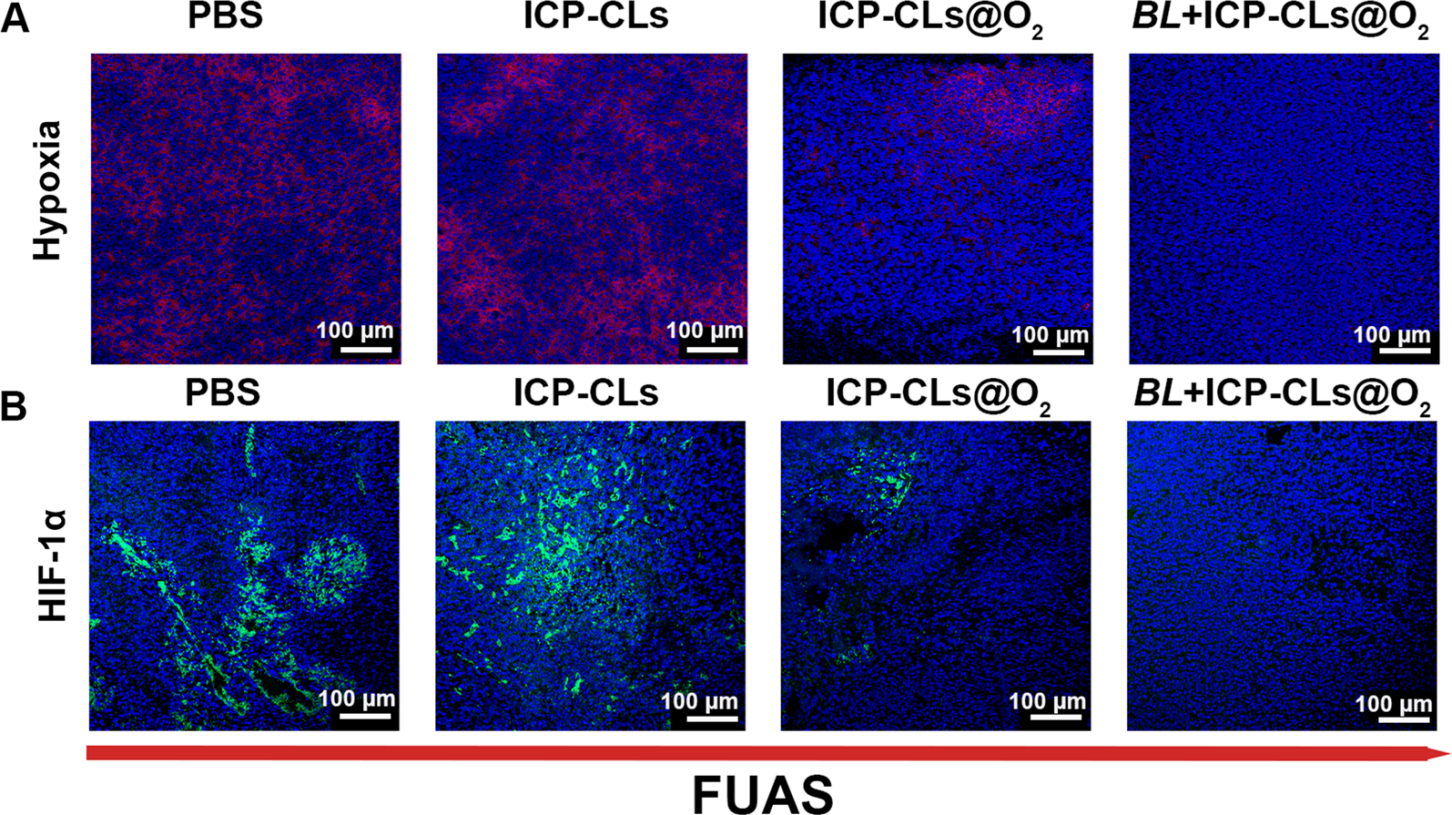
Evaluation of the ability to relieve hypoxia in vivo. **A** Immunofluorescence images of tumor slices incubated with anti-pimonidazole. **B** Immunofluorescence images of tumor slices incubated with HIF-1α antibody

The hypoxia regions appeared red after HypoxyprobeTM-1 Kit labeling, and the tumor nuclei appeared blue (Fig. 5A). After FUAS ablation, a large number of hypoxia regions were still visible in group PBS and group ICP-CLs, because neither group contained oxygen. Compared with these groups, the hypoxia zone of group ICP-CLs@O_2_ was reduced to some extent, but it was still more than that of group *BL* + ICP-CLs@O_2_. These results indicate that ICP-CLs@O_2_ can successfully release oxygen in the body under ultrasonic stimulation, alleviating hypoxia in tumor target areas. However, the *BL* + ICP-CLs@O_2_ group targeting ability was superior to group ICP-CLs@O_2_ 's due to the presence of *BL*. As a result, more ICP-CLs@O_2_ were maintained in the tumor target area, and more oxygen was released, improving the oxygen production impact.

Tumor resistance to several tumor treatments, such as chemotherapy, radiation, and PDT, is closely correlated with hypoxia. Hypoxia, particularly for chemotherapy, frequently leads to MDR and makes chemotherapy less effective by sequentially upregulating HIF-1, the MDR1 gene, and P-glycoprotein [21]. The reduction of the hypoxic environment will cause HIF-1 to be down-regulated, preventing tumor resistance. As shown in Fig. 5B, the expression trend of HIF-1α also proves that bio-targeted oxygen production probes can effectively relieve hypoxia and down-regulate the expression of HIF-1α. The above results indicate that bio-targeted oxygen production probes can effectively alleviate tumor drug resistance by improving tumor hypoxia and down-regulating the expression of HIF-1α, thus improving the efficacy of chemotherapy.

### Dual-modality imaging in vitro

**Fig. 6:**
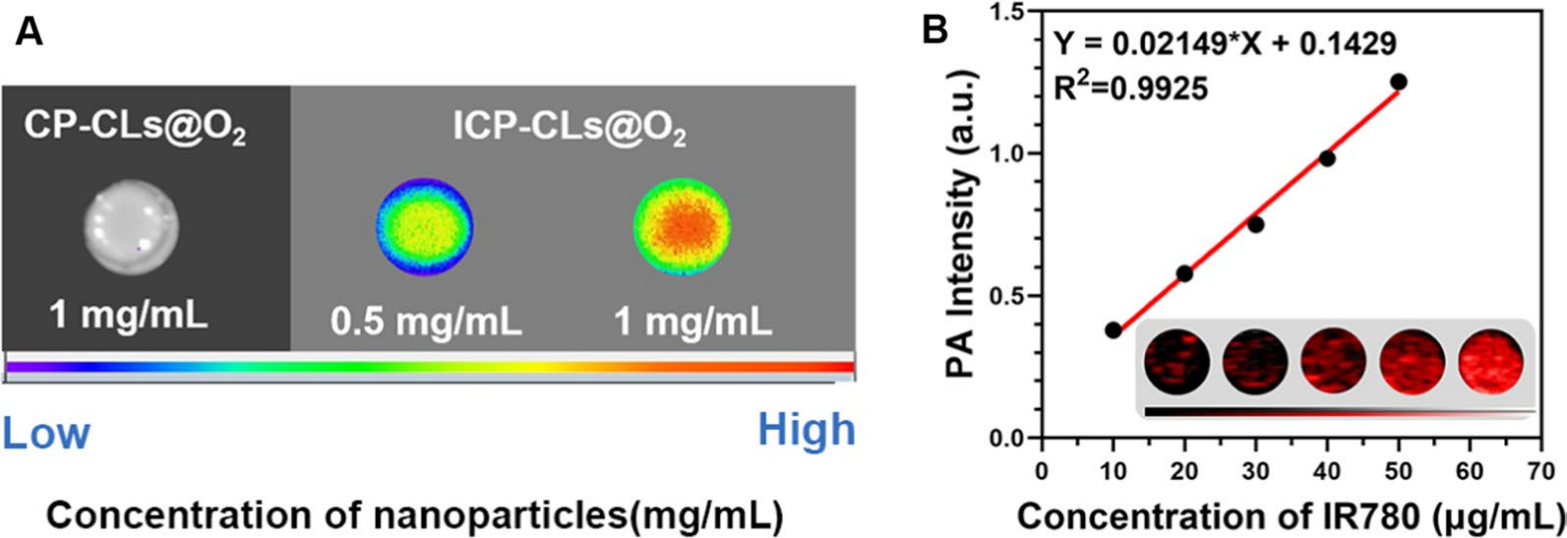
Dual-modality imaging of multi-functional oxygen-producing NPs (ICP-CLs@O_2_) in vitro. **A** FL images of CP-CLs@O_2_ and ICP-CLs@O_2_. **B** The PA image of different concentrations ICP-CLs@O_2_, and the linear relationship between the PA signal value with the concentration

As shown in Fig. 6A, compared with CP-CLs@O_2_ without IR780, ICP-CLs@O_2_ has good fluorescence imaging ability, and fluorescence intensity is related to concentration. The fluorescence imaging capability of ICP-CLs@O_2_ lays a foundation for the subsequent dynamic detection of the distribution of bio-targeted oxygen production probes in vivo. Since the PA signal strength and the concentration of ICP-CLs@O_2_ are well correlated (Fig. 6B), this suggests that ICP-CLs@O_2_ performs well in PA imaging. If bio-targeted oxygen production probes were used in vivo, PA imaging should be able to gather functional biological data at tumor locations.

### Dual-modality imaging in vivo

**Fig. 7:**
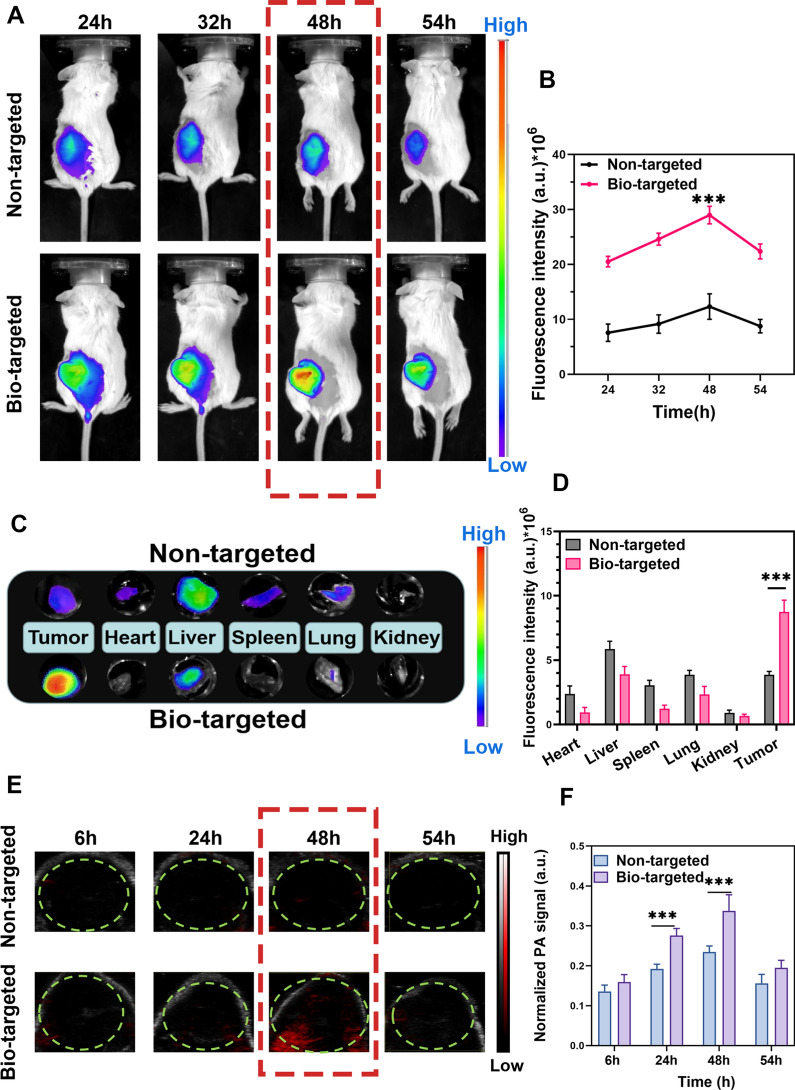
Dual-modality imaging of bio-targeted oxygen production probes in vivo. **A** FL imaging and metabolic distribution of bio-targeted oxygen production probes in vivo. **B** Quantitative FL intensity of tumor tissue at 24 h, 32 h, 48 h, 54 h. **C** Biodistribution of bio-targeted oxygen production probes in tumor and significant organs. **D** Quantitative FL signal of bio-targeted oxygen production probes in tumor and major organs. **E** PA imaging and **F** quantitative PA intensity of tumor after injection of bio-targeted oxygen production probes at 6 h, 24 h, 48 h, 54 h

FL imaging was used to identify the biological distribution of probes in vivo. As shown in Fig. 7A, ICP-CLs@O2 displayed good fluorescence imaging capabilities in vivo. Since IR780 effectively targets tumor mitochondria, both groups can benefit from targeted imaging [51]. Quantitative analysis and comparison (Fig. 7B) revealed that the FL intensity of the tumor in the bio-targeted group was much higher than in the non-targeted group without *BL* (***P < 0.001). Due to the fact that *BL* used electrostatic adsorption to direct much ICP-CLs@O_2_ to remain in the tumor and extended the ICP-CLs@O_2_ stay at the tumor location. Group bio-targeted combines the tumor targeting capabilities of IR780 and biological targeting vector *BL.* The FL signal peaked 48 h after injection, indicating that the enrichment of probes at the tumor site was greatest at this time, which is vital for subsequent in vivo treatment. To further observe the distribution of probes in the organs, in vitro fluorescence imaging was performed on the organs (Fig. 7C). The non-targeted group had the highest concentration of ICP-CLs@O_2_ in the liver. Because reticuloendothelial phagocytes play a crucial role in NPs metabolism (Fig. 7D). The ICP-CLs@O_2_ in the bio-targeted group, however, were concentrated mainly in the tumor as a result of *BL* guidance.

PA imaging results showed that the bio-targeted group probes had good tumor-targeting PA imaging ability (Fig. 7E), and the trend was consistent with fluorescence imaging. The signal intensity also peaked 48 h after injection (Fig. 7F). The bio-targeted oxygen production probes in vivo use enhance the treatment's effectiveness and safety by lowering the dispersion of NPs in healthy tissues.

The above results indicate that bio-targeted oxygen production probes have FL and PA tumor-targeting imaging capabilities. Dual-modality imaging combined with tumor diagnosis and treatment can provide more comprehensive information, improve the effectiveness and safety of treatment, and integrate the advantages of dual-modality imaging.

### Synergistic effect of bio-targeted oxygen production probes with FUAS

**Fig. 8:**
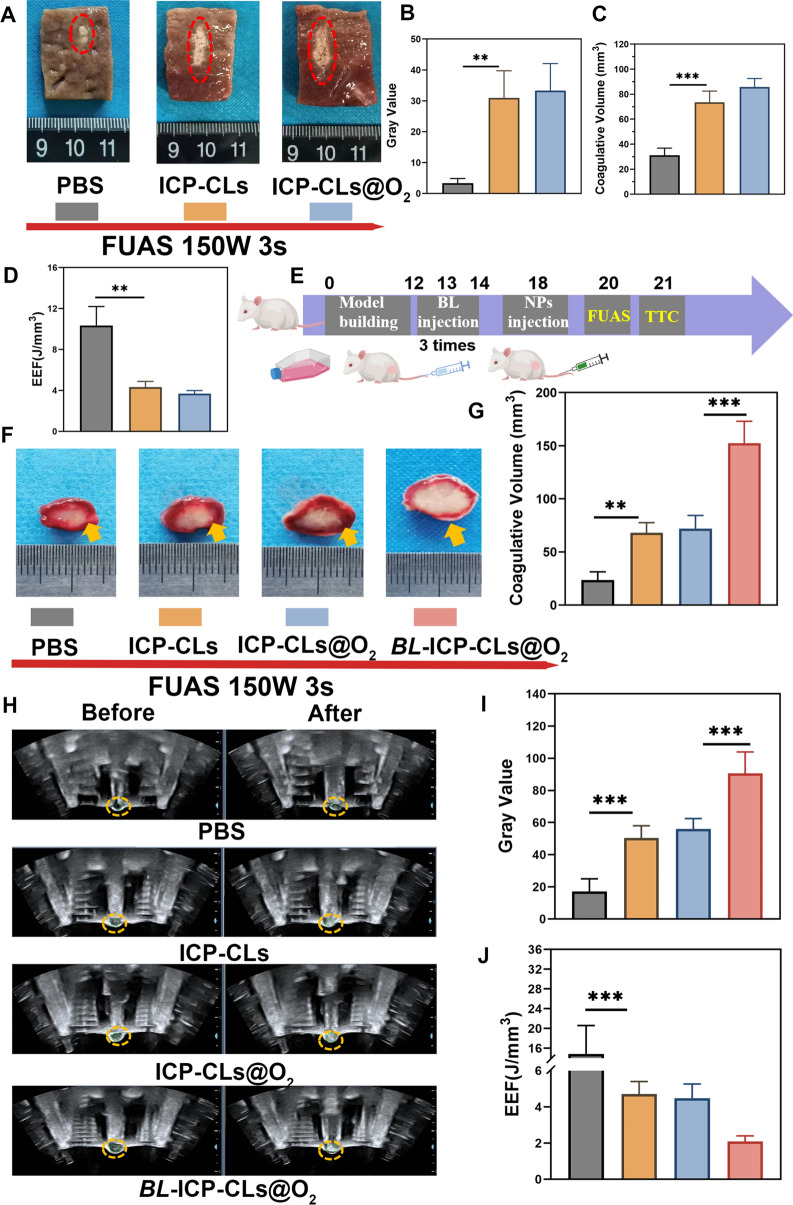
Synergistic effect of bio-targeted oxygen production probes with FUAS. **A** The in vitro ablated bovine liver image (PBS, ICP-CLs, ICP-CLs@O_2_). **B**–**D** Quantitative analysis of grayscale value, necrosis volume, and EEF. **E** Diagram of FUAS treatment synergistic by bio-targeted oxygen production probes in vivo. **F** TTC labeling revealed tumor coagulative necrosis following FUAS. **G** Statistical analysis of coagulative necrosis volume. **H** US image before and after FUAS. **I**, **J** Quantitative analysis of grayscale value and EEF at the target area

The results of bovine liver ablation showed that the liver was grayish white after ablation, the normal tissue without ablation was purplish red (Fig. 8A). Both the gray level and the coagulative necrosis volume of the ICP-CLs and ICP-CLs@O_2_ groups were more obvious than those of the PBS group (**P < 0.01), indicating that both ICP-CLs and ICP-CLs@O_2_ could significantly enhance FUAS ablation (Fig. 8B, C). Both ICP-CLs and ICP-CLs@O_2_ contain PFH, which can induce liquid–gas phase transition after excitation by focused ultrasound and enhance efficacy. The ablation efficiency can be reflected by the emergency efficiency factor (EEF), and the higher the ablation efficiency, the smaller the value. Figure 8D shows that the ICP-CLs and the ICP-CLs@O_2_ had significantly lower EEFs than the PBS group (**P < 0.01). The results of in vitro bovine liver ablation experiments all proved that ICP-CLs@O_2_ could be used as a synergist in FUAS treatment.

Figure 8E depicts the strategy for the in vivo experiment. After TTC staining, the coagulated necrotic area of tumor tissue became more evident and appeared white (Fig. 8F). Compared with PBS group, other groups showed different degrees of FUAS enhancement, among which group *BL* + ICP-CLs@O_2_ was the most significant (Fig. 8G). The change of grayscale (Fig. 8H, I) and EEF analysis also showed the same trend (Fig. 8J). The results of in vivo ablation experiments indicated that the bio-targeted oxygen production probes could be used as a synergist to enhance FUAS therapy significantly. PFH could cause a cavitation effect in FUAS ablation to destroy tumor cells because of its phase transition characteristics. *BL*, as a biological targeting carrier for tumors, can greatly improve the concentration of synergists at the tumor site, and the combination of the two effects achieves biological targeting synergism. Meanwhile, the FUAS ablation was conducted under dual-mode imaging, and the appropriate treatment time was chosen to achieve the most effective ablation result.

### FUAS-Chemo combined antitumor therapy in vivo

**Fig. 9:**
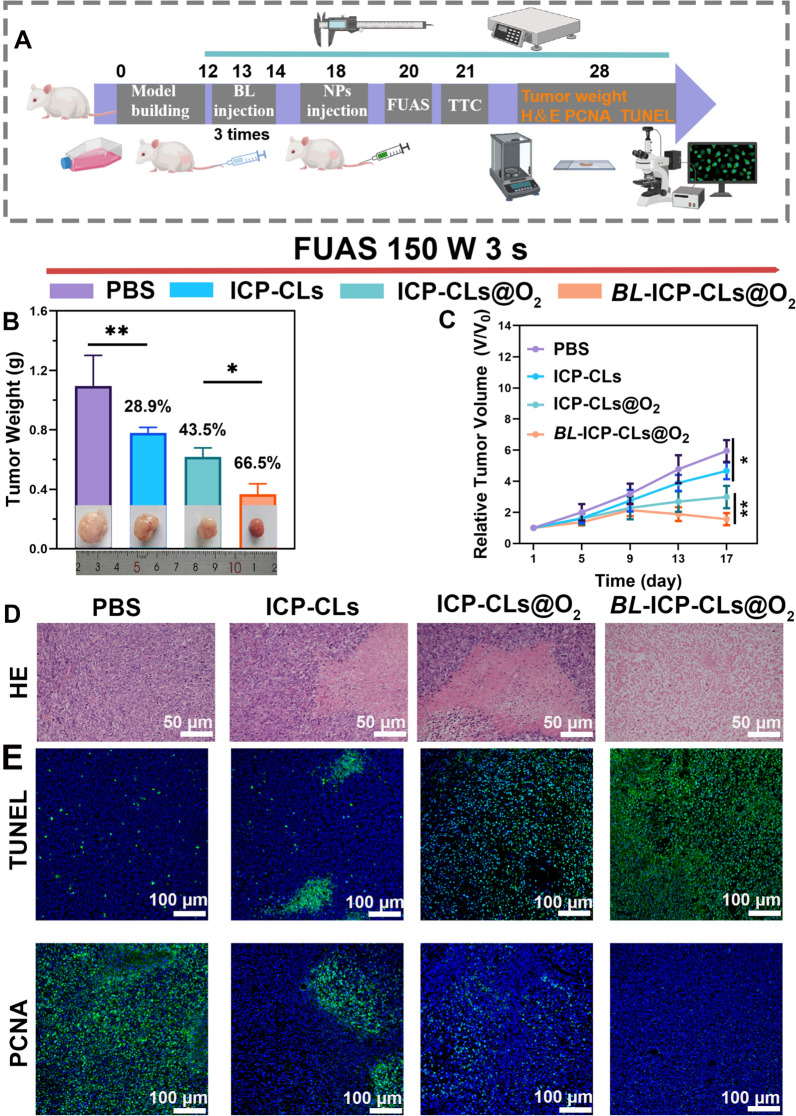
FUAS-Chemo combined antitumor therapy in vivo. **A** Diagram of FUAS-Chemo combined antitumor therapy in vivo. **B** Tumor weight and digital photos of different groups (PBS, ICP-CLs, ICP-CLs@O_2,_ *BL* + ICP-CLs@O_2_) on the 17th day. **C** Analysis of Relative tumor volume with PBS, ICP-CLs, ICP-CLs@O_2,_ *BL* + ICP-CLs@O_2_ groups. **D** H&E staining image of tumor tissues after treatment. **E** TUNEL and PCNA immunofluorescence images of tumor tissues after therapy

Figure 9A depicts the strategy for the in vivo antitumor treatment. As shown in Fig. 9B, the *BL* + ICP-CLs@O_2_ group had the smallest tumor volume, the lightest tumor weight, and the highest tumor growth inhibition value, indicating that the *BL* + ICP-CLs@O_2_ group had the most apparent tumor growth inhibition effect. The changes in relative tumor volume were continuously observed and plotted. The growth trend of the tumor in group *BL* + ICP-CLs@O_2_ was the slowest, which reflected an excellent tumor inhibition effect (Fig. 9C). Compared with the PBS group, the ICP-CLs group also inhibited tumor growth, however, the hypoxia zone at the tumor tissue and the lack of targeting limited the effect of chemotherapy. Although oxygen alleviated hypoxia at the tumor site in group ICP-CLs@O_2_, the chemotherapy effect was still inferior to that in group *BL* + ICP-CLs@O_2_. Because bio-targeted oxygen production probes not only achieved biological tumor targeting, but also degraded hypoxia at the tumor site, improved the drug chemotherapy effect, and realized the effective combination of FUAS-Chemo therapy. Immunohistochemical results also showed a similar trend (Fig. 9D, E). H&E staining results showed that, except for group PBS, other groups showed obvious nuclear pyroptosis and nucleolysis, among which group *BL* + ICP-CLs@O_2_ was the most significant. Apoptotic cells are labeled green in TUNEL fluorescence staining. In PCNA fluorescence staining, tumor-proliferating cells appear green. The area of apoptotic cells in each group ranged from small to large (PBS group, ICP-CLs group, ICP-CLs@O_2_, *BL* + ICP-CLs@O_2_ group). Moreover, the area of proliferative cells in each group ranged from large to small (PBS group, ICP-CLs group, ICP-CLs@O_2_, *BL* + ICP-CLs@O_2_ group). No matter whether PCNA or TUNEL staining, bio-targeted oxygen production probes showed a good therapeutic effect.

### Biosafety assessment

The safety of the bio-targeted oxygen production probe is a prerequisite for treatment. Additional file 1: Fig. S9 showed that the viability of HUVECs treated with ICP-CLs@O_2_ at various concentrations showed that ICP-CLs@O_2_ were safe. During the whole treatment process, mice in group *BL* + ICP-CLs@O_2_ showed a slight weight loss after *BL* injection and then recovered to normal soon after, with their weight fluctuating within the normal range, indicating that the bio-targeted oxygen production probes-based treatment strategy would not affect the normal growth of mice (Fig. S10). Blood biochemical and routine blood indexes showed that the indexes of the treatment group fluctuated within the normal range, and there was no significant difference between the treatment group and the control group (Additional file 1: Fig. S11). The results of H&E staining indicated that there was no apparent pathological damage to the major organs, suggesting that the bio-targeted oxygen production probes had good biological safety to the normal tissues and organs (Additional file 1: Fig. S12).

## Conclusion

Bio-targeted oxygen production probes have been developed to address the limitations of existing tumor therapies, such as poor targeting, single diagnostic and therapeutic image patterns, and drug resistance caused by hypoxia. The probe has the following benefits: (1) The existence of the hypoxic zone limits the effect of tumor treatment. However, unexpected effects can be achieved by using this feature and selecting the appropriate vector. The selection of probiotic *bifidobacterium* as the tumor target carrier has good biological safety. The target is not a specific protein, but the characteristic hypoxic region of solid tumors has certain universality and is suitable for most solid tumors. (2) The introduction of IR780 endows the bio-targeted oxygen production probes with FL and PA imaging capabilities. With the addition of two imaging modes, the original ultrasonic monitoring can be enriched, and drug delivery can be visualized. Dual-mode imaging is essential for tumor diagnosis and treatment. (3) PFH can not only carry oxygen but also enhance FUAS treatment. Meanwhile, FUAS has good tissue penetration, which can accurately control PFH to release more oxygen. By alleviating the hypoxia in the tumor area, improving the efficacy of chemotherapy, and preventing tumor recurrence and metastasis. (4) CBP, a commonly used chemotherapy drug in clinical therapy, can destroy residual tumors and achieve antitumor treatment of FUAS. It is wrapped in liposomes, enriched in the tumor target area guided by bacteria, and released after being stimulated by FUAS, reducing the damage to normal tissues, which can not only improve the effectiveness of treatment but also ensure the safety of treatment.

The cooperation of bio-targeted oxygen production probes and FUAS therapy is anticipated to address the drawbacks of current therapies, improve efficacy while ensuring the safety of treatment, and provide a unique approach to clinical therapy.

## Supplementary Information

**Additional file 1**: **Fig. S1.** Light microscope image of ICP-CLs@O_2_ (400 magnification). **Fig. S2.** The standard curve of IR780. **Fig. S3.** The standard curve of CBP. **Fig. S4.** In vitro release curves of CBP. **Fig. S5.** Particle size of ICP-CLs@O_2_ over a period of 13 days. **Fig. S6.** Zeta potential of ICP-CLs@O_2_ over a period of 13 days **Fig. S7.** The hemolysis test of ICP-CLs@O_2_ at different concentrations. **Fig. S8.** Flow cytometry analysis binding between *Bifidobacterium longum* and DiI-labeled ICP-CLs@O_2_. **Fig. S9.** Cell viability assay of different concentration ICP-CLs@O_2_ incubated with HUVECs. **Fig. S10.** Variation trend of body weight in different groups of mice. **Fig. S11.** Hematological assay of BALB/c mice. **Fig. S12.** H&E staining of major organs from the control group and the treated groups.

## Data Availability

All data needed to support the conclusions are present in the paper and/or the Additional file 1. Additional data related to this study are available from the corresponding authors upon reasonable.
