# Supplementary material for: Dual mode imaging guided multi-functional bio-targeted oxygen production probes for tumor therapy
Source: J Nanobiotechnology. 2023 Apr 29;21:142. doi: 10.1186/s12951-023-01901-7 (PMC10148460; doi:10.1186/s12951-023-01901-7)
Supplement: Supplementary file 1 — Additional file 1: Fig. S1. Light microscope image of ICP-CLs@O2 (400 magnification). Fig. S2. The standard curve of IR780. Fig. S3. The standard curve of CBP. Fig. S4. In vitro release curves of CBP. Fig. S5. Particle size of ICP-CLs@O2 over a period of 13 days. Fig. S6. Zeta potential of ICP-CLs@O2 over a period of 13 days Fig. S7. The hemolysis test of ICP-CLs@O2 at different concentrations. Fig. S8. Flow cytometry analysis binding between Bifidobacterium longum and DiI-labeled ICP-CLs@O2. Fig. S9. Cell viability assay of different concentration ICP-CLs@O2 incubated with HUVECs. Fig. S10. Variation trend of body weight in different groups of mice. Fig. S11. Hematological assay of BALB/c mice. Fig. S12. H&E staining of major organs from the control group and the treated groups. [file 12951_2023_1901_MOESM1_ESM.doc]

**Additional for**

Dual mode imaging guided multifunctional bio-targeted oxygen production probes for tumor therapy

*Yaotai Wang^1,2^, Zhong Zhang^1^, Li Ren^1^, Yong Luo^1^, Qi Wang^1,2^, Jianzhong Zou^*1,2^*

1. State Key Laboratory of Ultrasound in Medicine and Engineering, College of Biomedical Engineering, Chongqing Medical University, Chongqing, 400016, China.

2. Chongqing Key Laboratory of Biomedical Engineering, Chongqing Medical University, Chongqing, 400016, China.

**Keywords**

Bacteriotherapy, Tumor hypoxia, Focused ultrasound ablation surgery, Dual mode imaging, Anti-tumor therapy


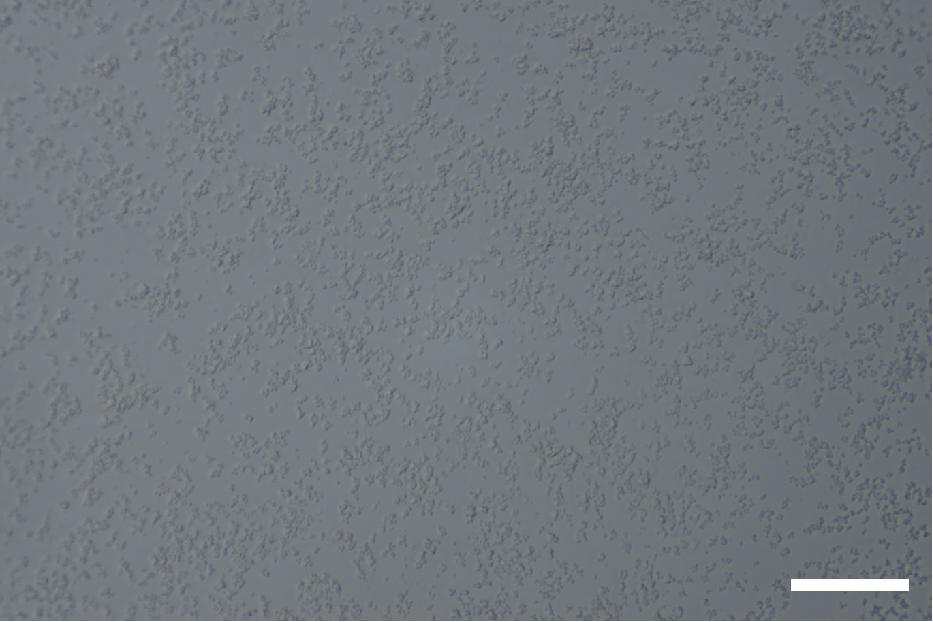


**Fig. S1** Light microscope image of ICP-CLs@O_2_ (400 magnification).


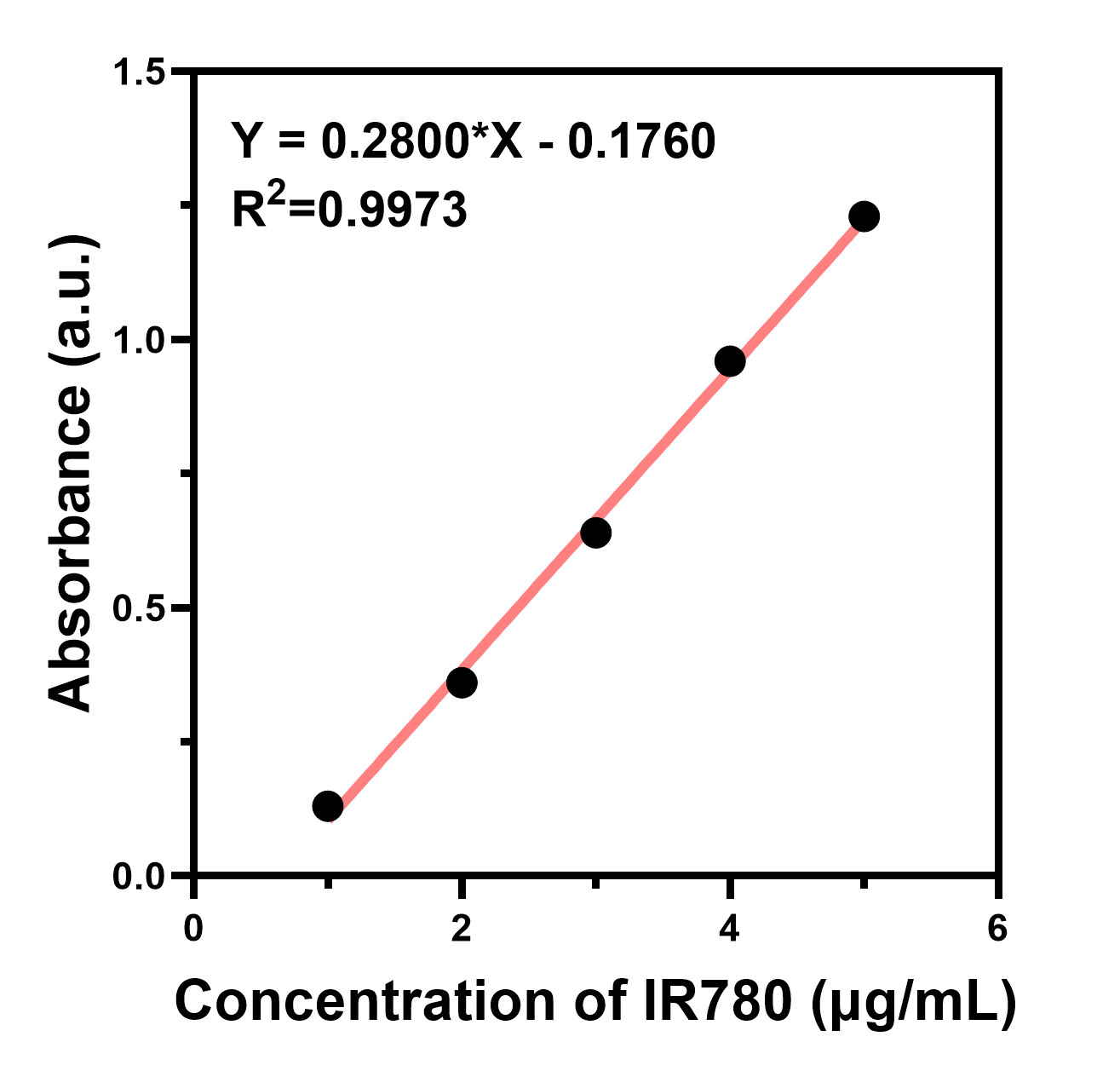


**Fig. S2** The standard curve of IR780.


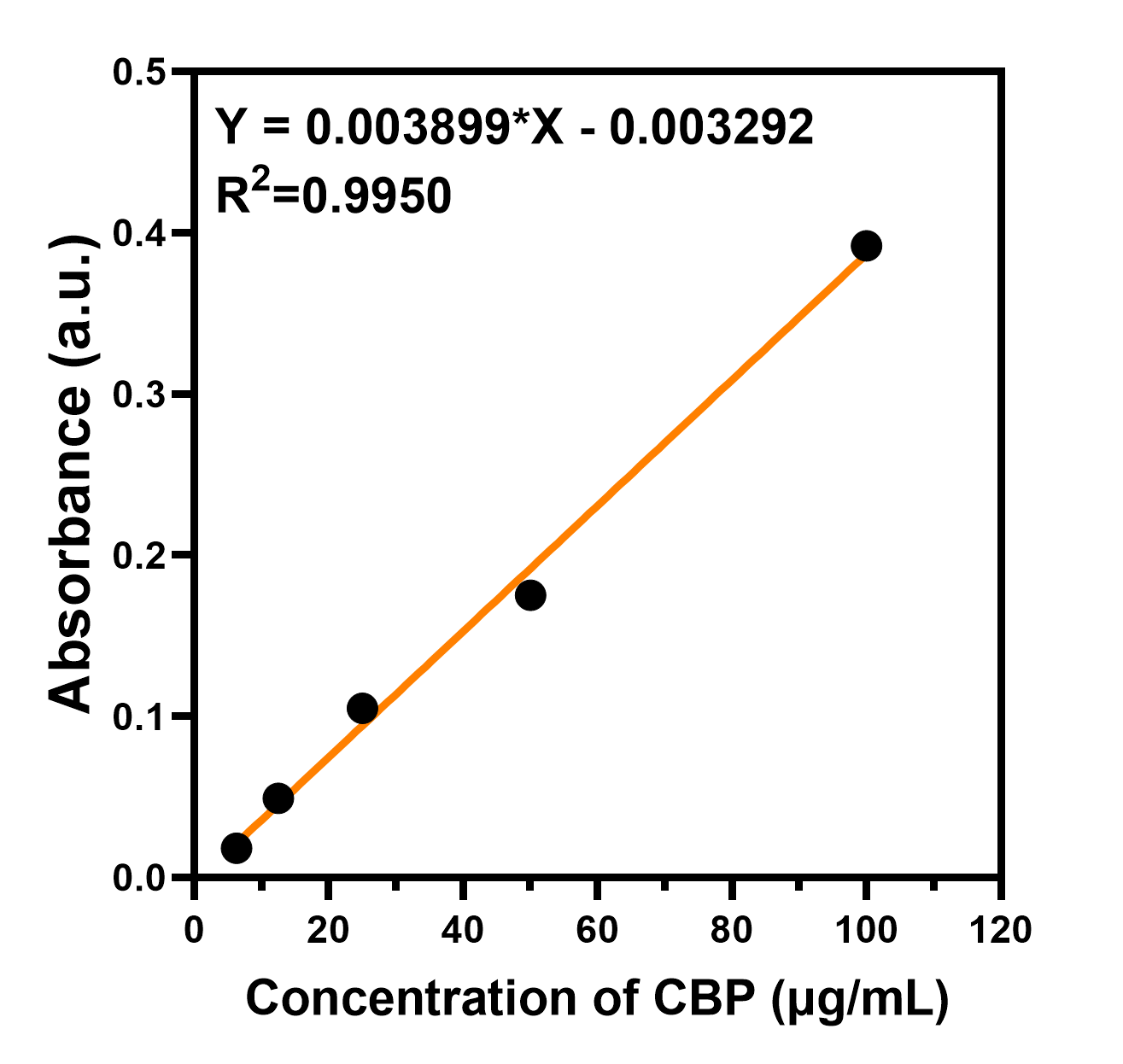


**Fig. S3** The standard curve of CBP.


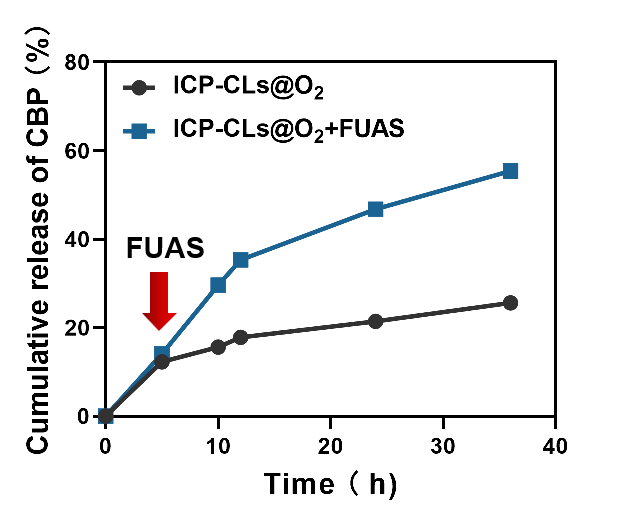


**Fig. S4 *In vitro* release curves of CBP**.


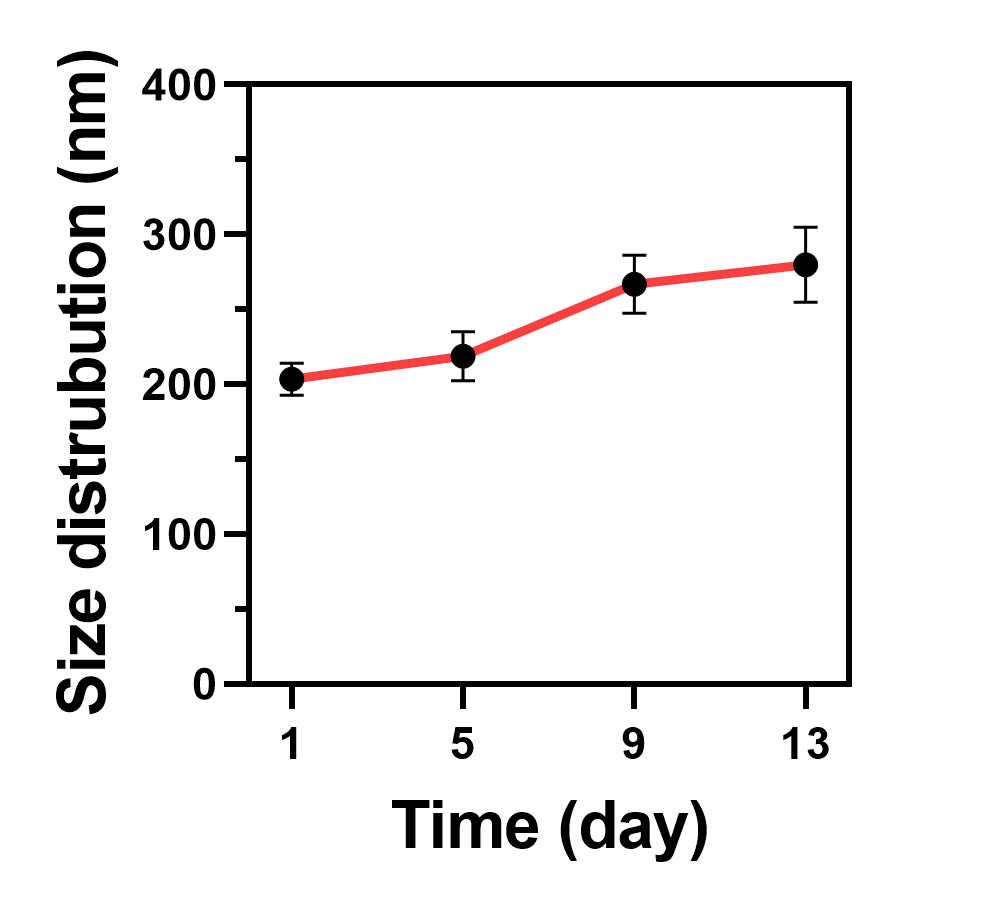


**Fig. S5** Particle size of ICP-CLs@O_2_ over a period of 13 days


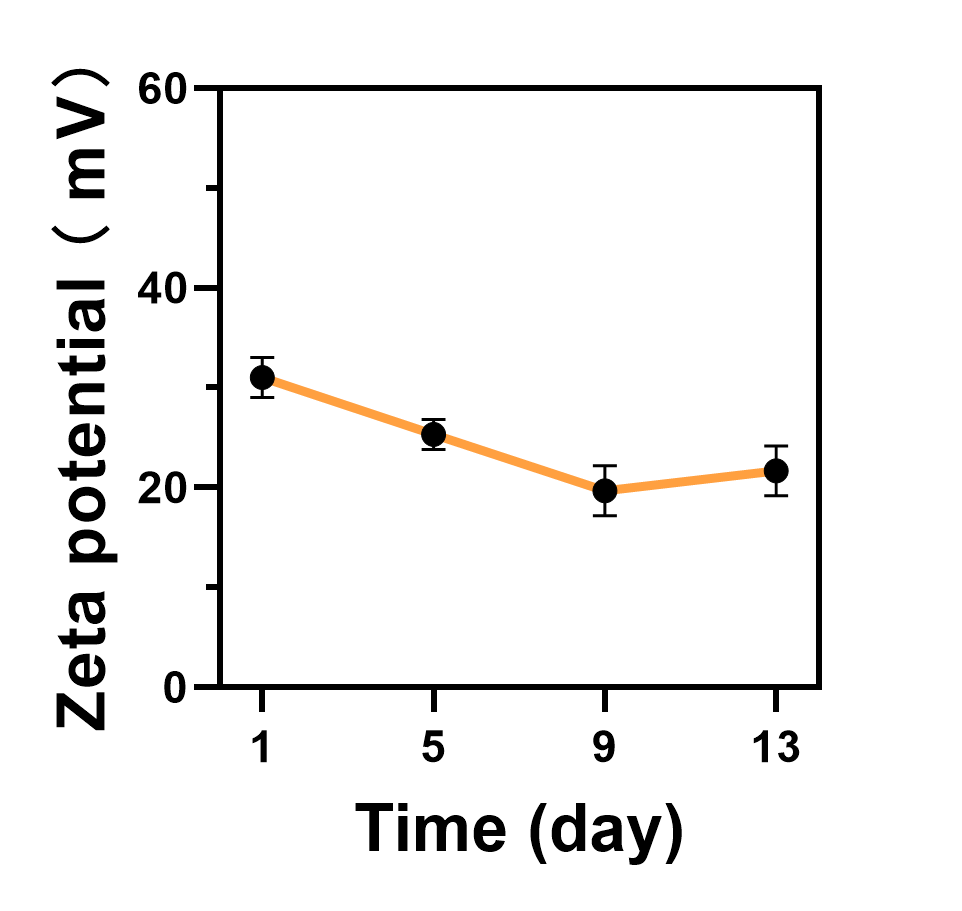


**Fig. S6** Zeta potential of ICP-CLs@O_2_ over a period of 13 days


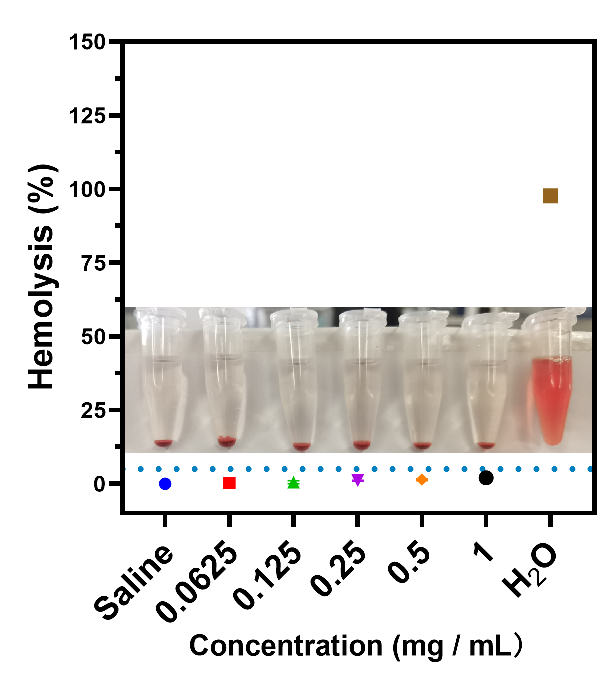


**Fig. S7** The hemolysis test of ICP-CLs@O_2_ at different concentrations.


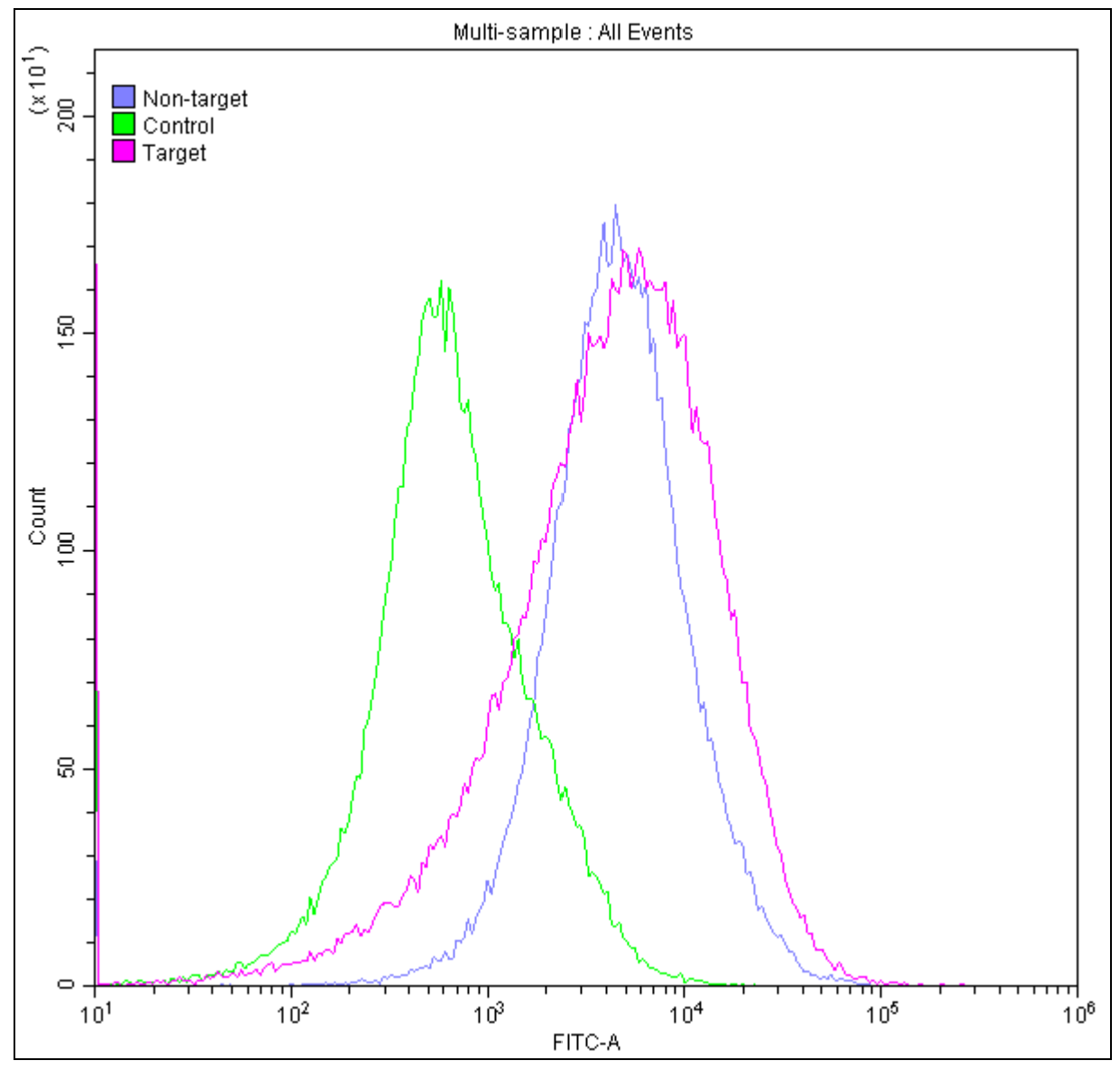


**Fig. S8** Flow cytometry analysis binding between *Bifidobacterium longum*

and DiI-labeled ICP-CLs@O_2_.


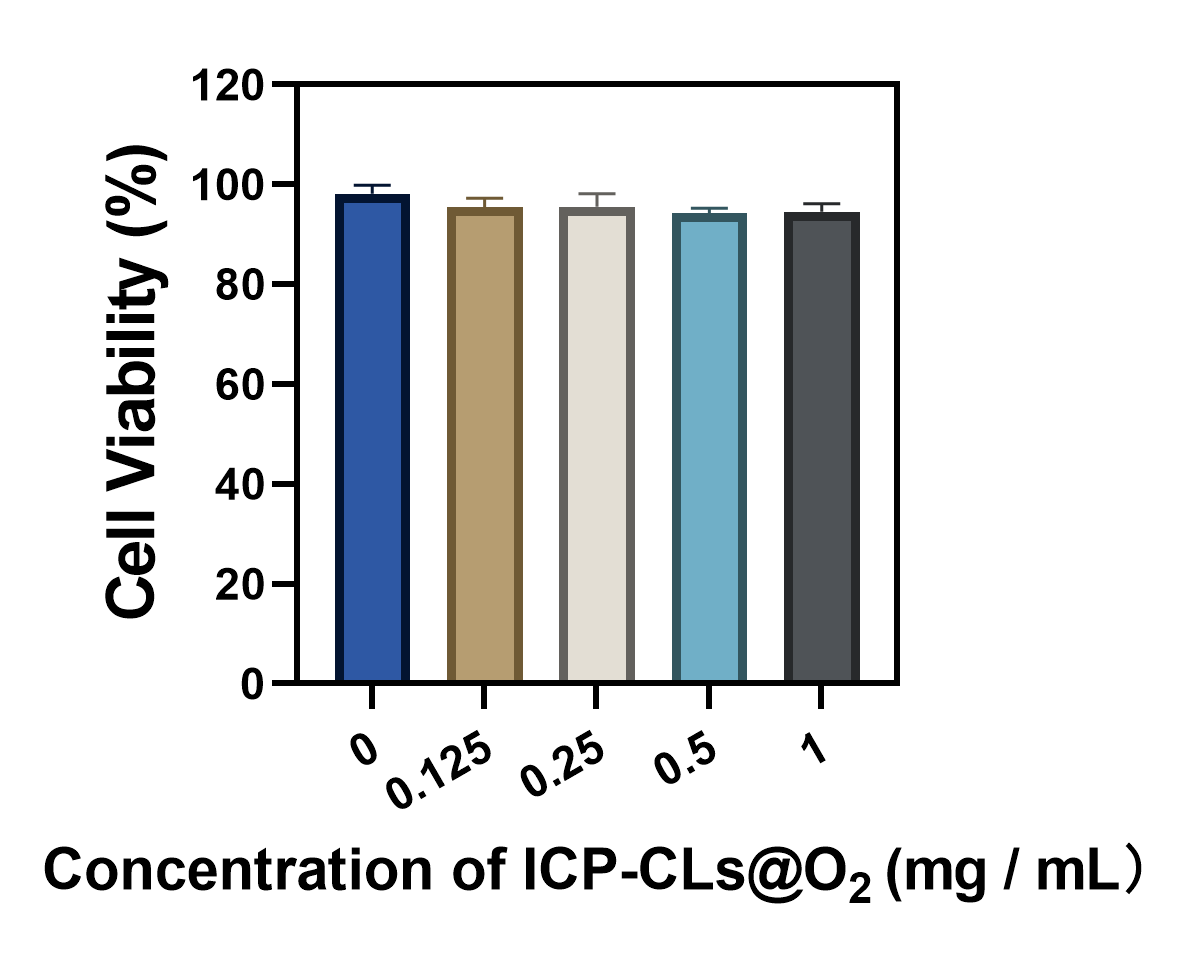


**Fig. S9** Cell viability assay of different concentration ICP-CLs@O_2_ incubated with HUVECs.


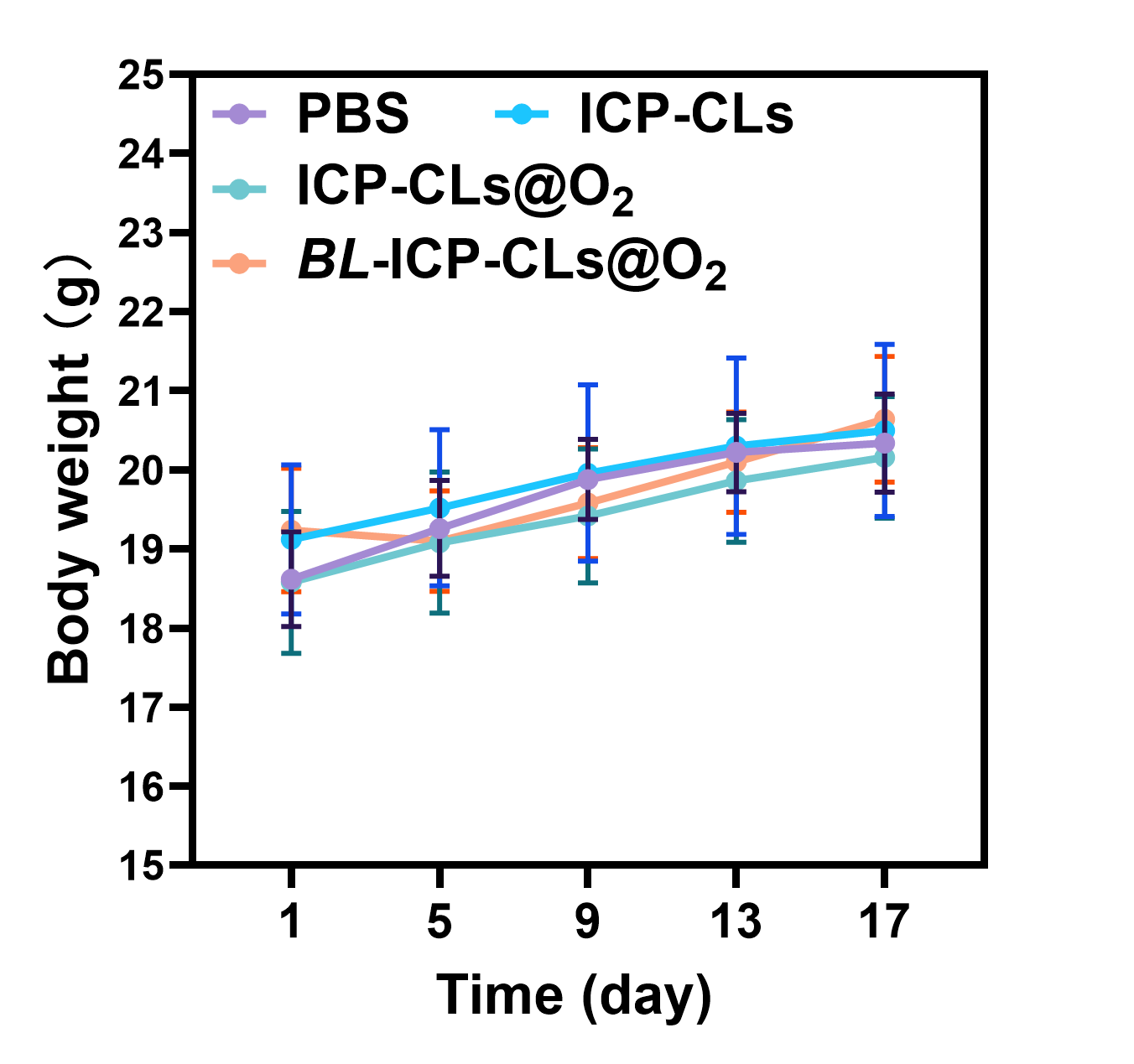


**Fig. S10** Variation trend of body weight in different groups of mice.


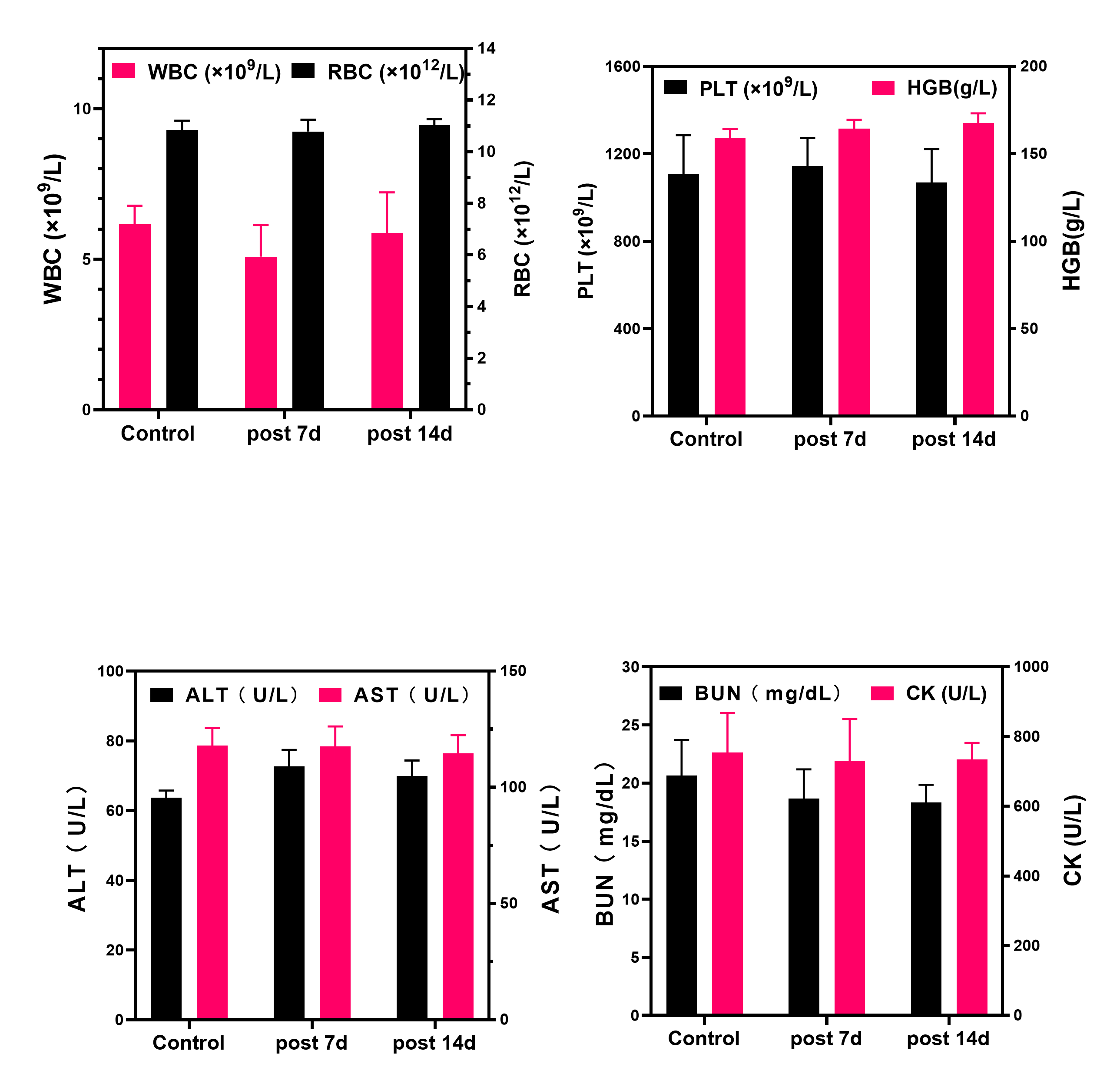


**Fig. S11** Hematological assay of BALB/c mice .


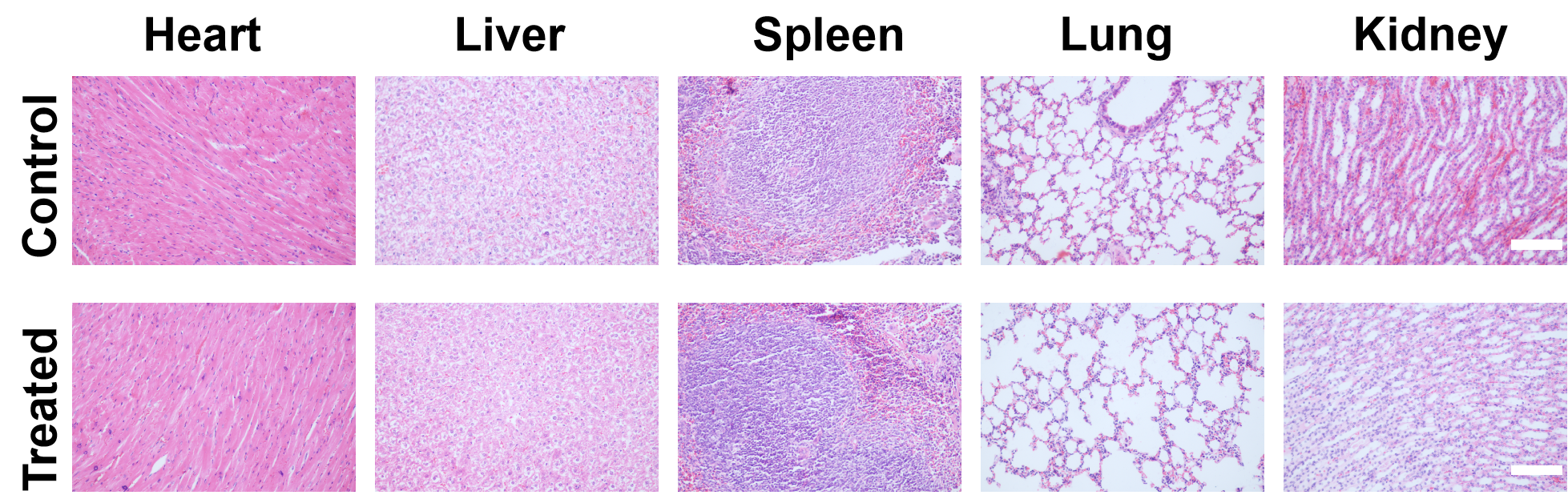


**Fig. S12** H&E staining of major organs from the control group and the treated groups, the scale bar is 50 μm.
